# Genomic Insight Into *Lacticaseibacillus paracasei* SP5, Reveals Genes and Gene Clusters of Probiotic Interest and Biotechnological Potential

**DOI:** 10.3389/fmicb.2022.922689

**Published:** 2022-06-16

**Authors:** Despoina Eugenia Kiousi, Christos Efstathiou, Konstantinos Tegopoulos, Ioanna Mantzourani, Athanasios Alexopoulos, Stavros Plessas, Petros Kolovos, Maria Koffa, Alex Galanis

**Affiliations:** ^1^Department of Molecular Biology and Genetics, Faculty of Health Sciences, Democritus University of Thrace, Alexandroupolis, Greece; ^2^Department of Agricultural Development, Democritus University of Thrace, Orestiada, Greece

**Keywords:** *Lacticaseibacillus paracasei*, probiotics, whole-genome sequence, comparative genomics, adhesion capacity, confocal microscopy, biotechnological potential

## Abstract

The *Lacticaseibacillus paracasei* species is comprised by nomadic bacteria inhabiting a wide variety of ecological niches, from fermented foodstuffs to host-associated microenvironments. *Lc. paracasei* SP5 is a novel strain, originally isolated from kefir grains that presents desirable probiotic and biotechnological attributes. In this study, we applied genomic tools to further characterize the probiotic and biotechnological potential of the strain. Firstly, whole genome sequencing and assembly, were performed to construct the chromosome map of the strain and determine its genomic stability. *Lc. paracasei* SP5 carriers several insertion sequences, however, no plasmids or mobile elements were detected. Furthermore, phylogenomic and comparative genomic analyses were utilized to study the nomadic attributes of the strain, and more specifically, its metabolic capacity and ability to withstand environmental stresses imposed during food processing and passage through the gastrointestinal (GI) tract. More specifically, Kyoto Encyclopedia of Genes and Genomes (KEGG) and Carbohydrate-active enzyme (CAZymes) analyses provided evidence for the ability of the stain to utilize an array of carbohydrates as growth substrates. Consequently, genes for heat, cold, osmotic shock, acidic pH, and bile salt tolerance were annotated. Importantly bioinformatic analysis showed that the novel strain does not harbor acquired antimicrobial resistance genes nor virulence factors, in agreement with previous experimental data. Putative bacteriocin biosynthesis clusters were identified using BAGEL4, suggesting its potential antimicrobial activity. Concerning microbe-host interactions, adhesins, moonlighting proteins, exopolysaccharide (EPS) biosynthesis genes and pilins mediating the adhesive phenotype were, also, pinpointed in the genome of *Lc. paracasei* SP5. Validation of this phenotype was performed by employing a microbiological method and confocal microscopy. Conclusively, *Lc. paracasei* SP5 harbors genes necessary for the manifestation of the probiotic character and application in the food industry. Upcoming studies will focus on the mechanisms of action of the novel strain at multiple levels.

## Introduction

The amended *Lactobacillus* genus is comprised by more than 200 species and subspecies, organized in clades based on metabolic and phenotypic attributes (Zheng et al., 2015). Full genome sequencing has provided evidence for the vast heterogeneity of this taxonomic group, leading to its reclassification into 26 genera (Zheng et al., 2020). The *Lacticaseibacillus paracasei* (formerly *Lactobacillus paracasei*) species includes non-motile, non-spore-forming, rod shaped, facultative anaerobic lactic acid bacteria (LAB) that populate several niches, from fermented foodstuffs to host-associated microenvironments (Zheng et al., 2020). The versatility is imprinted in their genomes, as they code for genes mediating adaptation to both anaerobic and aerobic environments, as well as in plant, insect, and animal hosts (Hatti-Kaul et al., 2018). Their employment as starter cultures for novel dairy and non-dairy fermented products, such as white brined cheese (Terpou et al., 2018), rice-flour (D’Auria et al., 2021), cranberry juice (Mantzourani et al., 2019a) and pomegranate beverage (Mantzourani et al., 2020) and therefore their biotechnological importance has been well documented. Moreover, their role in the prevention or management of several intestinal and extraintestinal diseases, and their potential application as probiotics, have been described in several studies (Hill et al., 2018). For example, consumption of milk fermented with *Lc. paracasei* strain Shirota showed to alleviate constipation in depressed individuals (Otaka et al., 2021), whereas *Lc. paracasei* Lpc-37^®^ administration could modulate stress and anxiety, in healthy adults (Patterson et al., 2020). In parallel, mechanistic studies have shown that *Lc. paracasei* isolates may present antiproliferative (Saxami et al., 2017), immunomodulatory (Chondrou et al., 2020), antibiofilm and antimicrobial activities (Acurcio et al., 2020), in a strain-specific manner.

The development and application of genomic methodologies have contributed significantly to the knowledge and understating of the probiotic and biotechnological potential of novel LAB strains (Kiousi et al., 2021). Indeed, phylogenomics paired with comparative genomics facilitate the taxonomic classification of isolates, pinpoint putative probiotic markers, and provide information about their fermentation profile and niche preference (Pan et al., 2021; Surve et al., 2022). Furthermore, genome mining helps toward the identification of virulence phenotypes and resistance to common antibiotics, offering valuable insights into potential health hazards associated with their consumption (Wang et al., 2021a).

Potential probiotic microorganisms can be found as autochthonous or allochthonous in the mammalian GI tract. Indeed, genomic data showed that they have co-evolved with the animal host, undergoing selective pressure and extensive genome modifications to adapt to the gut mucosa and epithelium (Duar et al., 2017). Specifically, host-associated LAB strains have traded the ability to produce vitamins and amino acids, that can be readily provided by the host, for the capacity to code for proteins associated with stress survival and host interactions (Salvetti and O’Toole, 2017). In this context, genes encoding for bile acid hydrolases, proton pumps and proteolytic enzymes, are abundant in their genome (Arnold et al., 2018).

In order to be efficient, probiotic bacteria must adhere to epithelial cells of the host, at least transiently (Monteagudo-Mera et al., 2019). Adhesion is a complex process that is mediated by protein-protein or protein-polysaccharide interactions. More specifically, host-associated lactobacilli code for proteins that can participate in specific carbohydrate-binding interactions, or non-specific electrostatic and hydrophobic attachment to the gut niche (Monteagudo-Mera et al., 2019). Importantly, the cell wall of numerous LAB strains is decorated with pili, housekeeping proteins and glycolytic enzymes with moonlighting functions, major contributors to mucin attachment (Reunanen et al., 2012; Waśko et al., 2014). Several *in silico* studies have identified these genetic clusters in the genome of *Lc. paracasei* strains, strengthening the notion that they can interact with the gut niche, when consumed (Bäuerl et al., 2010; Koryszewska-Bagińska et al., 2019).

*Lc. paracasei* SP5 is a LAB strain, originally isolated from kefir grains (Mantzourani et al., 2019b). Preliminary evaluation, based on a series of established *in vitro* tests, including tolerance to bile salts, resistance to digestion enzymes and acidic pH, as well as susceptibility to common antibiotics, demonstrated that, *Lc. paracasei* SP5 presents probiotic potential (Mantzourani et al., 2019b). Recent studies from our lab have also shown that the novel strain possesses desirable biotechnological properties. Indeed, *Lc. paracasei* SP5 was successfully utilized for the production of fermented chokeberry juice (Bontsidis et al., 2021) and white brined cheese (Plessas et al., 2021). In the present study, genomic approaches were applied to further characterize the probiotic and biotechnological attributes of *Lc. paracasei* SP5. Firstly, whole genome sequencing and assembly, were performed to construct the chromosome map of the strain. The genomic stability of the strain was estimated based on the presence of plasmids and mobile elements. Average nucleotide identity (ANI) was used as a metric to confirm that the novel strain is unique, while comprehensive phylogenomic analysis was employed to validate its classification to the *Lc. paracasei* species. Moreover, comparative genomic analysis was performed to detect genetic loci related to resistance to extreme conditions, bile acid, low pH, osmotic and oxidative stress, and KEGG and CAZymes analyses to determine the metabolic profile of the novel strain. Annotation algorithms were employed to detect genetic clusters for bacteriocin production and biofilm formation, as well as genes implicated in the adhesive phenotype and the production of pili and exopolysaccharides. The adhesion properties of the novel strain were also studied and evaluated *in vitro*, employing a quantitative microbiological method and confocal microscopy.

## Materials and Methods

### Bacterial Strains, Culture Conditions and DNA Isolation

*Lc. paracasei* SP5 was previously isolated from kefir grains (Mantzourani et al., 2019b). *Lc. rhamnosus* GG ATCC 53103 (LGG) was acquired from DSMZ (Braunschweig, Germany). The bacterial strains were grown O/N in de Man, Rogosa and Sharp (MRS) broth (Condalab, Madrid, Spain) at 37°C, under anaerobic conditions. For DNA isolation, overnight cultures of *Lc. paracasei* SP5 were pelleted by centrifugation at 8,000 × g for 4 min. The pellet was lysed, and DNA was extracted using the NucleoSpin^®^ Tissue kit (Macherey-Nagel, Düren, Germany), according to manufacturer’s instructions. DNA fragmentation was determined in 1% (w/v) agarose gel, and the quantity and quality of the isolated nucleic acids were also determined spectrophotometrically at 260 nm using NanoDrop^®^ ND-1000 UV-Vis Spectrophotometer (Thermo Fisher Scientific, Waltham, MA, United States).

### Whole Genome Sequencing and *de novo* Assembly

The isolated genomic DNA was sequenced using the Illumina NovaSeq6000 (2 × 151 paired ends) platform. A total of 10,389,922 paired-end reads were obtained. The quality of the reads was determined using FASTQC (v0.11.9; Andrews, 2010), and low-quality reads were removed using Trimmomatic (version 0.39; Bolger et al., 2014). *De novo* assembly was executed with SPAdes (version 3.15.1), with default parameters, selecting the –careful parameter to minimize mismatches (Bankevich et al., 2012). Assembly metrics were calculated with the QUality Assessment Tool (QUAST; version 5.2.0; Gurevich et al., 2013).

### Genome Annotation

Genome annotation was performed using Prokka (version 1.14.5; Seemann, 2014), and the local version of Prokaryotic Genome Annotation Pipeline (PGAP; Tatusova et al., 2016), with default parameters. The presence of plasmids in the assembled sequence was determined using PlasmidFinder (Carattoli et al., 2014) and of mobile genetic elements with MobileElementFinder (Johansson et al., 2021). Prophage regions were detected using PHAge Search Tool Enhanced Release (PHASTER; Arndt et al., 2016) and insertion sequence elements with ISFinder (Siguier et al., 2006). The assembly was searched for the presence of Clustered Regularly Interspaced Short Palindromic Repeats (CRISPR) arrays using CRISPRDetect (version 2.4; Biswas et al., 2016) and PILER-CR (Edgar, 2007). The EggNOGmapper (version 2.0) tool of the online EggNOG database (version 5.0; Huerta-Cepas et al., 2019) was used for the classification of predicted proteins into Clusters of Orthologous Groups (COGs). BlastKOALA (version 2.2) was utilized for the assignment of proteins into KEGG Orthology (KO) groups and the production of KEGG maps (Kanehisa et al., 2016). Additionally, CAZyme annotation was performed using the dbCAN2 meta server (Zhang et al., 2018) and Traitar was used for phenotypic characteristics prediction (Weimann et al., 2016). The visualization of the genome assembly was completed using Artemis (version 18.1.0; Carver et al., 2012).

### Comparative Genomics

The genome sequences of all available *Lc. paracasei* strains (January 2022) were obtained using a python script and were categorized based on isolation source. ANI was calculated for all strains using Pyani, a python module (version 0.2.10; Pritchard et al., 2015) to verify that *Lc. paracasei* SP5 is a unique strain. Pangenome analysis of *Lc. paracasei* strains was performed with Roary (version 3.13.0; Page et al., 2015), and core genome sequences were used to construct a phylogenomic tree with FastTree 2.1 (Price et al., 2010). Whole genome sequences of 14 *Lc. paracasei* strains originating from dairy and non-dairy sources, LGG, *Lactiplantibacillus pentosus* BGM48, *Lactiplantibacillus plantarum* DF and *Staphylococcus aureus* NCTC 8325, were aligned with progressiveMauve (Darling et al., 2010). The publicly available online EMBL tool “Interactive Tree of Life” (iTol; version 6.1.1; Letunic and Bork, 2016) was used for the visualization of the trees.

### Detection of Genetic Elements Associated With Probiotic Characteristics

Genes involved in antimicrobial resistance were investigated using Resistance Gene Identifier (RGI; version 5.2.0) and ResFinder 4.1 (Zankari et al., 2012; Bortolaia et al., 2020). Genes coding for virulence factors were determined with VirulenceFinder 2.0 (Joensen et al., 2014; Tetzschner et al., 2020), while putative pathogenic sequences were determined with PathogenFinder 1.1 (Cosentino et al., 2013). Putative bacteriocin clusters were identified using BAGEL4 (van Heel et al., 2018). The presence of proteins involved in the adhesion phenotype was investigated using BLAST+ (Basic Local Alignment Search Tool; Camacho et al., 2009) and alignment of genes of the *spaCBA* and *spaFED* clusters was performed with ClustalW (Thompson et al., 1994). Visualization of the alignment of *spaF* gene sequences was performed with Jalview (Waterhouse et al., 2009) and of the phylogenetic tree with the publicly available tool, iTol. Classification of protein families involved in the adhesion phenotype was performed by InterPro (Blum et al., 2021) and conserved domains were identified with Pfam (Mistry et al., 2021) and ScanProsite (de Castro et al., 2006). Characterization of the physicochemical properties of the putative *spaF* pilin was performed using ProtParam (Gasteiger et al., 2005). SignalP 6.0 was utilized to predict N-terminal signals (Teufel et al., 2022).

### Human Cancer Cell Line

The human colon adenocarcinoma cell line HT-29 was purchased from the American-Type Culture Collection (ATCC, LGC Standards, Middlesex, United Kingdom). Cells were maintained at 37°C, 5% CO_2,_ in a humidified atmosphere under sterile conditions in RPMI-1640 medium, supplemented with 10% heat-inactivated fetal bovine serum (FBS), 100 U/mL penicillin, and 100 μg/mL streptomycin (all from Thermo Fisher Scientific, Waltham, MA United States).

### Preparation of Viable Bacteria for Adhesion Assays

Strains *Lc. paracasei* SP5 and LGG were incubated O/N in MRS broth, at 37°C, under anaerobic conditions. The next day, the bacterial cells were centrifuged at 4,000 × g for 10 min. The cell pellet was resuspended in RPMI-1640 medium supplemented with 10% heat-inactivated FBS and 20 mM 4-(2-hydroxyethyl)-1-piperazineethanesulfonic acid (HEPES; all from Thermo Fisher Scientific) at a final concentration of 10^8^ CFU/mL.

### Assessment of Bacterial Adhesion by Quantitative Analysis

The quantitative adhesion assay was performed as described previously, with minor modifications (Plessas et al., 2020). HT-29 cells were seeded in 24-well plates at a density of 40 × 10^4^ cells per well and incubated for 14 days to form a monolayer. A day prior to the treatments, the cell monolayer was washed with phosphate buffered saline (PBS; Thermo Fisher Scientific) and fresh, antibiotic-free medium was added. The next day, viable *L. paracasei* SP5 or LGG cells, at a final concentration of 10^8^ CFU/mL were added to each well, with each strain being tested in duplicate. After 4 h of coincubation at 37°C, cells were washed twice with PBS and lysed with 1% Triton X-100 (Sigma-Aldrich, Saint Louis, MO, United States). The lysates were serially diluted in Ringer’s solution (Lab M, Lancashire, United Kingdom), plated on MRS agar, and incubated at 37°C, until the formation of visible colonies. Colony forming units per milliliter (CFU/mL) were determined with the formula: CFU/mL = (No. of colonies × dilution factor)/volume of culture plate and was used as viable count measure. The experimental procedure was repeated three independent times and the results are represented as mean ± standard deviation.

### Assessment of Bacterial Adhesion *via* Confocal Microscopy

Confocal microscopy was used for the visualization of bacterial attachment onto HT-29 cells. To this end, cells were seeded in a 6-well plate at a density of 5 × 10^5^ cells per well on No. 1.5 coverslips and were incubated overnight at 37°C with 5% CO_2_ in a humidified incubator. T he next day, cells were washed with PBS and were stained with Hoechst 33342 (Biotium, Hayward, CA, United States) for 20 min, according to manufacturer’s instructions. Simultaneously, lactobacilli were stained with 10 μM carboxyfluorescein succinimidyl ester (CFSE; ThermoFisher Scientific) in PBS to a final concentration of 10^8^ CFU/mL, for 20 min at 37°C. After staining, the lactobacilli suspension was co-cultured with HT-29 cells for three and a half hours at 37°C with 5% CO_2_ in a humidified incubator. Subsequently, cells were washed three times with fresh medium and were stained with CellBrite Red Cytoplasmic Membrane Dye (Biotium), according to manufacturer’s instructions. Then, cells were washed with fresh PBS and fixed in 4% paraformaldehyde (PFA; AppliChem, Darmstadt, Germany) in PHEM solution consisted of 25 mM HEPES, 10 mM EGTA (Merck Millipore, Burlington, MA, United States), 60 mM PIPES, 2 mM MgCl_2_ (Applichem) pH 6.9, for 12 min at room temperature, followed by three washes with PBS. Finally, coverslips were mounted in homemade mowiol 4-88 (AppliChem) medium.

Image acquisition was performed on a customized Andor Revolution Spinning Disk Confocal system (Yokogawa CSU-X1; Yokogawa, Tokyo, Japan), built around an Olympus IX81 (Olympus Shinjuku, Tokyo, Japan), with 40 × 0.95NA air lens (UPlanSApo; Olympus Shinjuku, Tokyo, Japan) and a digital camera (Andor Ixon+885; Andor Technology Ltd., Belfast, Northern Ireland). The system was controlled by Andor IQ3 software (Andor Technology). Images were acquired as z-stacks with a z-step of 1 μm, through the entire volume of the cells. For each image, maximum projection of z-stacks was generated, and background was subtracted using a custom script in ImageJ (National Institute of Health, United States). The number of HT-29 cells with adhered lactobacilli was counted manually with the Cell Counter plugin on ImageJ (version 1.53f51). For each condition, more than 1000 cells were counted in two independent experiments and the ratio of the HT-29 cells with adhered bacteria to the total number of eukaryotic cells were calculated.

## Results and Discussion

### Genome Features and Stability

**FIGURE 1:**
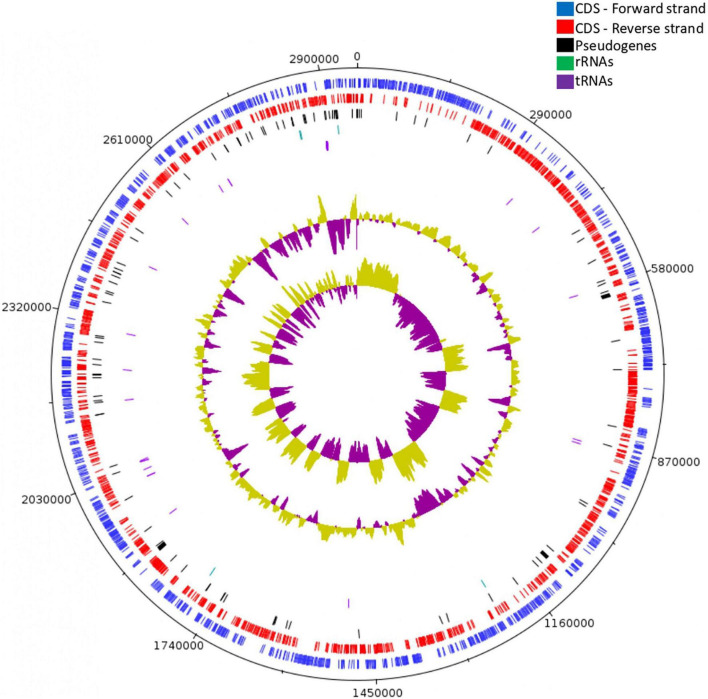
Circular genome map of *Lc. paracasei* SP5, constructed using Artemis. From outer circle to inner, genomic features are presented as: forward strand CDS (blue), reverse strand CDS (red), pseudogenes (black), rRNA genes (green) tRNA genes (purple), GC content and GC skew.

Whole-genome sequencing, *de novo* assembly and genome annotation were performed to investigate the genomic features of *Lc. paracasei* SP5 (Figure 1). The genome of *Lc. paracasei* SP5 has a total length of 2,958,982 bp and a GC content of 46.3%. The strain harbors 2,920 predicted genes; 2,870 coding DNA sequences (CDSs), 105 pseudogenes, 5 rRNA, 42 tRNA and 3 ncRNAs (Table 1). Genome metrics can be a crude indicator of the lifestyle of LAB strains. Indeed, studies have shown that genome size of lactobacilli ranges from 1.28 to 4 Mb, depending on their preferred environmental niche (Duar et al., 2017). Over the course of evolution, members of the *Lactobacillus sensu lato* have underwent a process of genome reduction, during the transition from free living to nomadic and matrix-associated bacteria (Sachs et al., 2011). Free-living and nomadic strains carry larger genomes (3–4 Mb) to support their survival in heterogenous matrixes. *Lc. paracasei* is a member of the former *Lc. casei* group alongside the nomadic *Lc. casei* and *Lc. rhamnosus* species (Hill et al., 2018). These species harbor genomes with median length and GC content of 2.9 Mb and 46–47%, respectively. They inhabit similar niches, predominantly dairy products, while they can also be found in association with the host (Hill et al., 2018). In this context, *Lc. paracasei* SP5 genome metrics may support its classification in nomadic lactobacilli. On the other hand, strictly host-associated strains, possess smaller genomes (1.28–3 Mb), resulting from extensive gene loss (Zheng et al., 2015; Duar et al., 2017). This phenomenon can be attributed to the fact that specializing to a nutrient-dense environment leads to the loss of redundant functions, such as amino acid biosynthesis, while strains become more selective in their energy sources (Fontana et al., 2018). In this context, *Lactobacillus iners* with a median genome length of 1.28 Mb is the most prominent example of extreme niche specialization, as it has acquired clusters for survival in the human vagina (Macklaim et al., 2011). The reverse process (gene accumulation) has been recorded in the *Limosilactobacillus fermentum* species, that is undergoing transition from host-associated to free living (Duar et al., 2017).

**TABLE 1:** Genome characteristics of *Lacticaseibacillus paracasei* SP5.

Genome characteristics	Value
Length	2,958,982 bp
GC content	46.3%
Total genes	2,920
CDSs	2,870
rRNAs	5
tRNAs	42
ncRNAs	3
Pseudogenes	105
No. of CRISPR arrays	0
Cas proteins	0
IS elements	122
**Phages**	
*Intact*	1
*Incomplete*	2
*Questionable*	2
**Antibiotic resistance genes**	
*Perfect hits*	0
*Strict hits*	0
*Loose hits*	202
Virulence genes	0
Probability of being a human pathogen	0.099
Plasmids	0

*Lc. paracasei* SP5 carries 122 insertion elements, 5 prophage regions (1 intact, 2 incomplete and 2 questionable; Supplementary Table 1) and no mobile genetic elements or plasmids (Table 1). Importantly, the strain lacks functional CRISPR arrays and does not code for Cas proteins, being therefore susceptible to bacteriophage assaults and accumulation of insertion sequences (Rath et al., 2015). Genome analysis with ISFinder showed that the majority of the insertion sequences originate from *Lc. casei* and *Lc. rhamnosus*, while several sequences were also derived from *Leuconostoc* and *Pediococcus* species. These bacteria are commonly found in the microbiome of dairy products, alluding to events of genetic transfer in these matrixes (Bonham et al., 2017). Insertion elements could play an important role in the expression of bacterial genes and genome evolution, and the availability of a large number of whole genome sequences have paved the way for the characterization of novel elements (Siguier et al., 2014). However, they can compromise genome stability, an important safety indicator for probiotics (Hill et al., 2014). Lactobacilli are known to possess a large number of transposons and mobile elements scattered in their genomes, providing evidence for transfer of genetic material in food and/or animal microbiota (Nicolas et al., 2007; Liu et al., 2009). Indeed, the intestinal cavity promotes these events in high frequencies (Lerner et al., 2017), and thus, it is of outmost importance to determine if they carry virulence factors and resistance genes that they can share with malignant species. On that note, bioinformatic analysis with RGI and VirulenceFinder showed that the novel strain does not harbor mobile antibiotic resistance genes or other virulence factors that could transport to bacteria of the gut microbiome. In this context, the probability of *Lc. paracasei* SP5 being a human pathogen is estimated to be below 0.1% (Table 1). Notably, *Lc. paracasei* strains have a long history of safe consumption, and previous studies have shown that administration in supplements or in fermented foodstuffs is well tolerated (Costa et al., 2014; Ren et al., 2022).

### Phylogenetic Analysis

**FIGURE 2:**
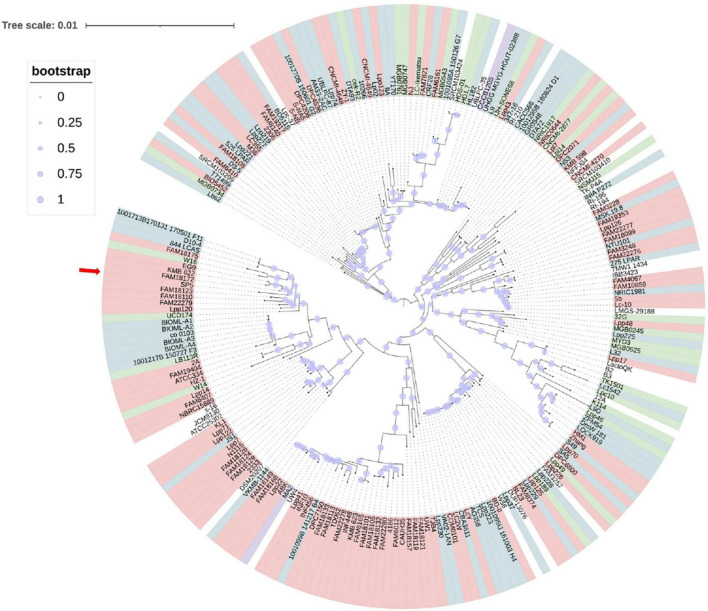
Approximately-maximum-likelihood phylogenetic tree of all available (239 as of January 2022) *Lc. paracasei* isolated from various sources (light pink – dairy products, blue – host-associated strains, green – vegeTables–associated strains, lilac – strains isolated from non-dairy beverages) based on orthologous genes calculated by Roary (version 3.13.0) and built with 1,000 bootstrap replications. The red arrow indicates the position of *Lc. paracasei* SP5 in the phylogenetic tree.

An approximately-maximum-likelihood phylogenetic tree based on orthologous gene clusters was built with 1,000 bootstrap replications to reveal the position of the novel strain within the *Lc. paracasei* species (Figure 2). Furthermore, a phylogenetic tree based on whole genome sequence of *Lc. paracasei* SP5 was, also, constructed, with *Staphylococcus aureus* NCTC8325 as an outgroup (Supplementary Figure 1). These findings agree with the preliminary classification of the strain to the *Lc. paracasei* species, based on 16S rRNA sequencing and species-specific multiplex PCR, with primers targeting the conserved *tuf* gene (Mantzourani et al., 2019b). Importantly, this is the first published work that presents a phylogenetic tree based on the core genome sequences of all available *Lc. paracasei* strains (as of January 2022), including their isolation source. As shown in Figure 2, *Lc. paracasei* SP5 clusters with other dairy isolates, as well as with one strain isolated from fermented soybeans, and several human-associated members of the species. The fact that strains do not form phylogenetic clusters based on their origin, reflects the nomadic character that is hardwired in their genomes (Duar et al., 2017). On a larger scale, the members of the former *Lactobacillus* genus, form clades based on their metabolic and fermentation capabilities, rather than their origin (Zheng et al., 2015). It is suggested that fermented foods are hardly the primary habitat of lactobacilli, however, tracing their origins is a challenging task (Duar et al., 2017).

**FIGURE 3:**
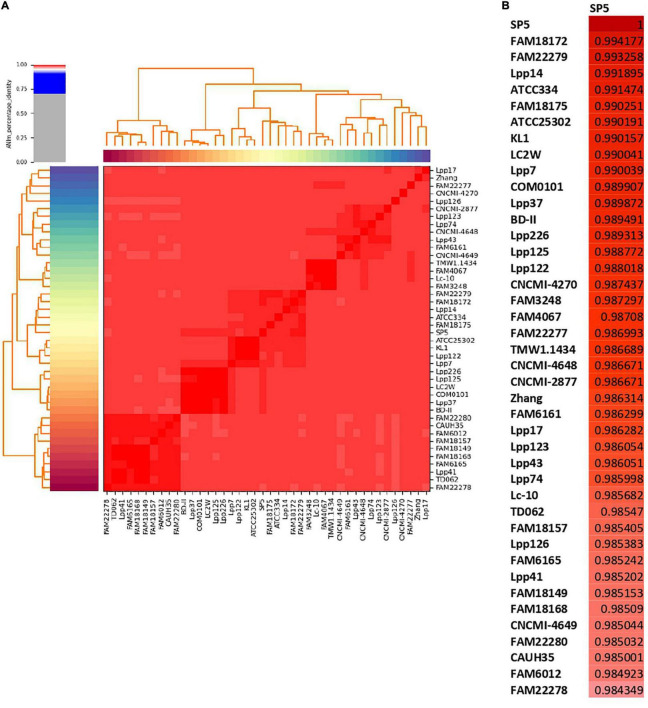
ANI of *Lc. paracasei* strains derived from fermented milk products estimated using Pyani (version 0.2.10). **(A)** ANI heatmap of all dairy isolates, **(B)** ANI of *Lc. paracasei* SP5 with dairy isolates.

ANI was selected as a metric for the nucleotide-level genomic similarity of *Lc. paracasei* SP5 with other strains of the species (Figure 3 and Supplementary Table 2), as well as for taxonomic classification with the cutoff for species boundaries set at 96% (Ciufo et al., 2018). In this study, ANI analysis was performed for all available strains showing that the novel strain is unique and presents high similarity with both dairy and non-dairy *Lc. paracasei* strains. More specifically, *Lc. paracasei* SP5 presents high genome similarity to *Lc. paracasei* Lpp120, *Lc. paracasei* KMB_622, *Lc. paracasei* FAM18172, all isolated from fermented milk products (Smokvina et al., 2013; Wüthrich et al., 2018; Figure 3B). Interestingly, *Lc. paracasei* SP5, also, presents high-level ANI (> 99%) to human-derived isolates, such as *Lc. paracasei* 844_LCAS (Roach et al., 2015), *Lc. paracasei* D10-4 and *Lc. paracasei* co_0103 (Jiang et al., 2019), as well as to *Lc. paracasei* W14 and W16, originally isolated from fermented soybeans (National Center for Biotechnology Information, 2017a,b) (Supplementary Table 2).

### Pangenome Analysis and Comparative Genomics

**FIGURE 4:**
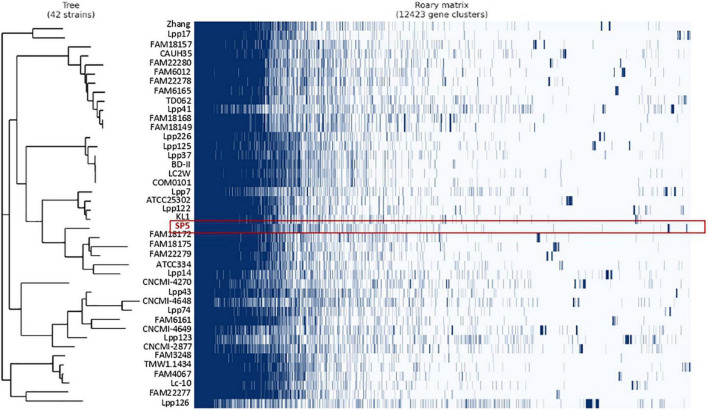
Pangenome analysis of *Lc. paracasei* strains isolated from fermented milk products, performed using Roary (version 3.13.0). The absence (white) or presence (dark blue) of core and accessory genes are depicted in the matrix. Highlighted in red are clusters assigned to *Lc. paracasei* SP5.

Pangenome analysis was performed in a subset of 42 strains derived from fermented milk products. The pangenome of these strains consists of a total of 12,423 orthologous groups (Figure 4). Amongst these, 489 genes belong to the conserved core genome, 741 to the soft-core genome, 2,596 to the shell genome and 8,596 genes to the cloud genome. The pangenome of these strains was further classified into COG categories (Table 2). The most represented category is “Function unknown” (17.11%), followed by “Replication and repair” (16.11%). *Lc. paracasei* SP5 codes for 97 unique, strain-specific gene groups that can be categorized into 15 COG categories. The majority of these strain-specific proteins possess unknown functions (19.05%) or are implicated in “Carbohydrate metabolism and transport” (16.67%). Further annotation revealed the presence of unique genes coding for transposases and for proteins involved in polysaccharide biosynthesis and transport (Supplementary Table 3). A previous study on a subset of 34 *Lc. paracasei* strains derived from a variety of sources, revealed that genes involved in microbe-host interactions can be found at the core genome of the species (Smokvina et al., 2013). Interestingly, genes mediating adhesion on the GI mucosa were also identified in the core genome of the dairy isolates included in this study. More specifically, genes coding for the moonlighting proteins with adhesive properties enolase (*eno*) and phosphoglycerate mutase (*pgm6*) were annotated in the core genome of the strains (Supplementary Table 4). It should be noted that the classification of pangenome proteins and proteins encoded by *Lc. paracasei* SP5 to COG categories followed a similar pattern (Table 2). However, the pangenome of the strains contains more proteins in the “Replication and Repair” (16.11%) category than *Lc. paracasei* SP5. This finding could be attributed to the fact that the vast array of transposable and insertion elements encoded by *Lc. paracasei* strains are classified into this category. In a previous pangenome comparative study of probiotic genera that included the emended *Lactobacillus* genus, it was found that the majority of proteins in the pangenome possess unknown functions, or cluster in the L category (Lukjancenko et al., 2012), in agreement with the pattern observed in the present study.

**TABLE 2:** Categorization of genes (SP5, Pangenome, SP5 unique genes) to clusters of orthologous genes (COGs).

Class	SP5	Pangenome	Unique
C, Energy production and conversion	111 (4.81%)	299 (3.17%)	1 (2.3%)
D, Cell cycle control and mitosis	38 (1.62%)	134 (1.42%)	0 (0%)
E, Amino Acid metabolism and transport	184 (7.83%)	539 (5.71%)	1 (2.38%)
F, Nucleotide metabolism and transport	111 (4.72%)	237 (2.51%)	0 (0%)
G, Carbohydrate metabolism and transport	231 (9.83%)	879 (9.32%)	7 (16.67%)
H, Coenzyme metabolism	66 (2.81%)	164 (1.74%)	2 (4.76%)
I, Lipid metabolism	57 (2.43%)	134 (1.42%)	0 (0%)
J, Translation	166 (7.06%)	306 (3.24%)	0 (0%)
K, Transcription	211 (8.98%)	632 (6.70%)	6 (14.29%)
L, Replication and repair	157 (6.68%)	1521 (16.11%)	5 (11.90%)
M, Cell wall/membrane/envelop biogenesis	125 (5.32%)	598 (6.34%)	4 (9.52%)
N, Cell motility	8 (0.34%)	29 (0.31%)	2 (4.76%)
O, Post-translational modification, protein turnover, chaperone functions	52 (2.21%)	139 (1.47%)	0 (0%)
P, Inorganic ion transport and metabolism	132 (5.62%)	450 (4.77%)	1 (2.38%)
Q, Secondary Structure	26 (1.11%)	72 (0.76%)	1 (2.38%)
T, Signal Transduction	52 (2.21%)	155 (1.64%)	1 (2.38%)
U, Intracellular trafficking and secretion	45 (1.91%)	144 (1.53%)	2 (4.76%)
V, Defense mechanisms	74 (3.15%)	365 (3.87%)	1 (2.38%)
S, Function Unknown	502 (21.36%)	1614 (17.11%)	8 (19.05%)
No category, General function prediction only	269 (11.45%)	1019 (10.81%)	5 (11.9%)
Total	2350 (100%)	9430 (100%)	42 (100%)

### Functional Classification of Genes and Prediction of the Metabolic Potential of *Lc. paracasei* SP5

**FIGURE 5:**
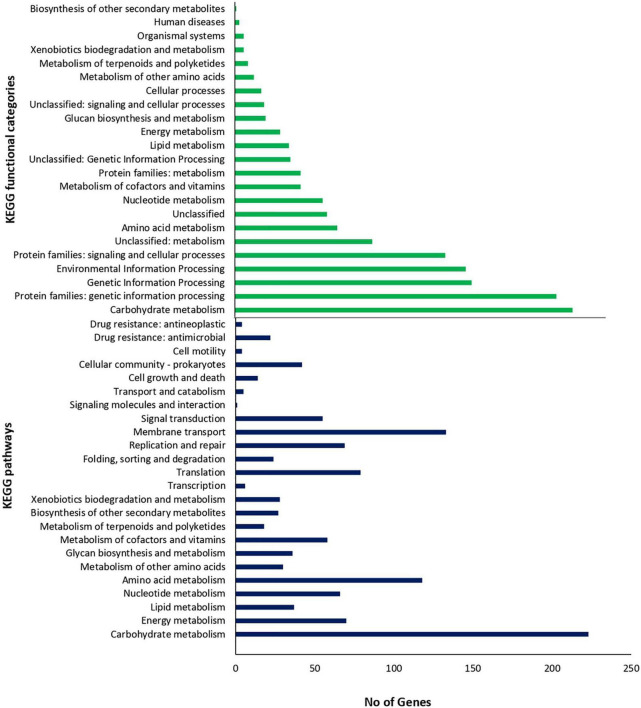
Number of proteins assigned to KEGG functional categories and pathways.

To gain a better insight into the functional characteristics of the genome of *Lc. paracasei* SP5, its protein sequences were allocated into the 20 COG categories using EggNog. As shown in Table 2, the most represented category is “Function Unknown” (21.36%), followed by “Carbohydrate metabolism and transport” (9.83%), “Transcription” (8.98%), “Translation” (7.06%) and “Replication and repair” (6.68%). KEGG analysis of the assembled genome resulted in protein assignment into 191 pathways, organized into 24 broader groups, and 23 functional categories (Figure 5). Similarly, to the COG profile of *Lc. paracasei* SP5 genes, most proteins were assigned to the “Carbohydrate metabolism” category (223 proteins), followed by the “Membrane transport” (133 proteins) and “Amino acid metabolism” (118 proteins) categories (Figure 5). Concerning KEGG pathway assignment, the majority of annotated proteins cluster in the “Biosynthesis of secondary metabolites” (162 proteins, KO 01110) and the “Microbial metabolism in diverse environments” (95 proteins, KO 01120) pathways (Figure 5). Complete biosynthetic pathways for the production of 3 (threonine, lysine and proline) out of 20 amino acids (Supplementary Figures 2–4), and incomplete clusters with one block missing for the production of cysteine, methionine, arginine, histidine, tryptophan and glutathione were, also, found in the genome of *Lc. paracasei* SP5. This finding could be indicative of the gene decay that the strain underwent during its evolutionary history. More specifically, loss of amino acid biosynthetic capability is a shared characteristic of strains transitioning from a free-living to a matrix-associated lifestyle (Duar et al., 2017). Importantly, *Lc. paracasei* SP5 codes for numerous proteolytic enzymes, peptidases (e.g., membrane dipeptidase, *pepD2*), oligopeptidases (e.g., oligopeptidase F, *pepF2*) and amino acid transporters, including the Di-/tripeptide transporter (*dtpT*), compensating for its limited biosynthetic capability. Recently, we have reached similar conclusions for the nomadic strains *Lp. pentosus* L33 and *Lp. plantarum* L125 (Stergiou et al., 2021; Tegopoulos et al., 2021). The proteolytic capability of strains to be incorporated in fermented foodstuffs could be an asset in the functional food industry, due to degradation of allergens or the production of bioactive metabolites after amino acid catabolism (Wang et al., 2021b). However, casein degradation could negatively affect the quality of fermented dairy products (Lesme et al., 2020). In this context, Traitar analysis showed that *Lc. paracasei* SP5 cannot break down this protein (Supplementary Figure 5), a finding supporting its application in the production of dairy foodstuffs. Additionally, amino acid catabolism could lead to the accumulation of biogenic amines in the food matrix (Wang et al., 2021b). Biogenic amines are commonly found in fermented products in low quantities, without necessarily affecting their quality and organoleptic characteristics (Li et al., 2018), however, their concentration should be monitored. The enzymes implicated in their production are decarboxylases and deiminases. PGAP annotation showed that *Lc. paracasei* SP5 codes for ornithine decarboxylase, an enzyme that catalyzes the production of putrescine from ornithine.

CAZymes analysis combined with KO assignment was used to decipher the ability of the strain to encode glycolytic and carbohydrate-binding modules and enzymes (Supplementary Table 5). More specifically, *Lc. paracasei* SP5 codes for 69 genes that can be further classified into four classes: 31 glycoside hydrolase (GH) genes, 32 glycosyltransferase (GT) genes, 3 carbohydrate-binding modules (CBMs), 3 carbohydrate esterase (CE) genes, supporting the catabolism of a broad range of carbohydrates, including glucose, mannose, glycogen, chitin. Traitar analysis also showed that it can utilize sucrose, maltose, D-mannose, malonate, citrate and possibly lactose, as growth substrates (Supplementary Figure 5). Furthermore, biosynthetic clusters for the production of chitin, cellulose, and lipopolysaccharides were also identified (Supplementary Table 5). KEGG, CAZymes and Traitar analysis showed that it heavily relies on carbohydrate metabolism, possessing full pathways for glycolysis through the Embden-Meyerhof pathway, gluconeogenesis, pyruvate oxidation and the pentose phosphate pathway. These metabolic attributes are characteristic of homofermentative strains, that produce lactic acid as the major byproduct of glycolysis (Duar et al., 2017). In this context, COG annotation showed that *Lc. paracasei* SP5 codes for D-lactate dehydrogenase that catalyzes the formation of D-lactic acid from pyruvate. Interestingly, it can also degrade galactose, and possibly starch, alluding to the fact that this strain could be used in both dairy and vegetable fermentation. These findings support the lack of niche specialization of the strain, revealing its ability to grow in a great variety of nutrient-dense environments, in agreement with previous data from our lab, where *Lc. paracasei* SP5 was successfully incorporated in cheese and fermented juice products (Bontsidis et al., 2021; Plessas et al., 2021). The promiscuity of the strain is a very useful tool for functional food industry, as it enables novel application in dairy and non-dairy food products. Accordingly, another presumed nomadic strain, *Lc. paracasei* K5, originally isolated from dairy products, was utilized to produce a pomegranate beverage (Mantzourani et al., 2020) and sourdough bread (Mantzourani et al., 2019c). Strain specific differences in the metabolic capacity of other members of the *Lc. paracasei* species also supports their use as starter cultures for green table olives (Sisto and Lavermicocca, 2012) or short ripened Caciotta-type cheese (Bancalari et al., 2020).

*Lc. paracasei* SP5 also contains genes that participate in the production of a plethora of vitamins and co-factors, however, most of these modules are incomplete (Supplementary Figures 6–9). The strain carries a full biosynthetic cluster for the production of C10-C20 isoprenoids, through the mevalonate pathway and can produce intermediates for diterpenoid, carotenoid and indole diterpene alkaloid biosynthesis. This pathway is scattered amongst eukaryotes and prokaryotes, playing an important role in the production of isoprenoids, the largest family of organic compounds that is comprised by quinones, hormones and other signaling molecules (Hoshino and Gaucher, 2018). Previously, a gene cluster for the mevalonate pathway was identified in *Lactobacillus helveticus* (Smeds et al., 2009). The ability of the *Lc. paracasei* SP5 to produce isoprenoids and other secondary metabolites of biological importance should be further characterized *in vitro* and *in situ.*

### Genome-Wide Analysis of Loci Conferring Probiotic and Biotechnological Potential

Genome annotation and comparative bioinformatical analysis were utilized for the detection of sequences that could be implicated in resistance to stress conditions, prevalent during industrial processing and digestion. Specifically, genes conferring tolerance to heat and cold shock, such as the cold shock proteins (Csp)A, B, C, and members of the HSP20 family, were pinpointed in the genome of the novel isolate (Table 3). Accordingly, *Lc. paracasei* SP5 codes for the GroEL/GroES chaperonin system assisting protein folding in extreme conditions. Based on the genetic attributes of the strain, it could also be resistant to osmotic shock, as it codes for response elements and osmoprotectant proteins. In greater detail, *Lc. paracasei* SP5 harbors genes for the production of glycine betaine binding factors (*opuAC, opuCC, choS*) and transporters (*gbuA, B*), an osmolyte that is accumulated in bacterial cells under hypertonic shock (Boch et al., 1996). No biosynthetic clusters for the production of glycine betaine were identified, suggesting that the strain may depend on extracellular supply. Additionally, the synergistic activity of GrpE with DnaK and DnaJ, during hyperosmotic shock could remedy possible damages in the macromolecular machinery of the cell (Schroder et al., 1993). It should be noted that in a transcriptomic study, *Lc. paracasei* SMN-LBK exhibited resistance to ethanol, that was accompanied by the induction of phosphofructokinase (PFK), GAPDH, and glycerol kinase (GK; Guo et al., 2020), also annotated in the genome of the novel strain. This finding could suggest that, *Lc. paracasei* SP5 could survive in beverages with low alcoholic content, however, this should be further investigated *in situ*. Furthermore, survival in saline media (6.5% NaCl) was predicted by the Traitar tool (Supplementary Figure 5). Physicochemical stress, including heat, high pressure, and osmotic shock, is a common strategy employed by the food industry to minimize the proliferation of human pathogens (Burgess et al., 2016). Strains of the former *Lc. casei* group generally present high tolerance to these conditions, supporting their application as starter or non-starter cultures in the food industry (Reale et al., 2015). Furthermore, *Lc. paracasei* SP5 harbors an effective oxidative stress response system (Table 3) that can support survival and damage repair in aerobic conditions during production. More specifically, the strain codes for peroxidases and NADH oxidases, as well as for redox-regulated molecular chaperones. *Lc. paracasei* strains, additionally, present another mechanism for oxidative stress resistance, manifested by the intracellular accumulation of manganese (Nierop Groot et al., 2005). The genetic cluster (*mtsCBA*) involved in this phenotype is also encoded by *Lc. paracasei* SP5, regulating the production of ABC-type manganese transporters. This cluster was shown to play an important role in the survival of *Lc. paracasei* strain Shirota in aerobic conditions (Serata et al., 2018). Apart from the innate ability of probiotic strains to cope with these conditions, additional strategies to guarantee survival of starter cultures have been adapted in the food industry. These commonly include the addition of oxygen-consuming enzymes or antioxidants, encapsulation or the modification of the food matrix to include prebiotics (Feng and Wang, 2020).

**TABLE 3:** Annotation of genes coded by *Lc. paracasei* SP5 that are implicated in stress response and host-microbe interactions.

locus tag	Gene function	Gene	E-value
***Gastrointestinal tract survival and stress response***			
SP5_000699	Penicillin-binding protein	*pbpX*	0.0
SP5_001309	Penicillin-binding protein	*mrdA*	0.0
SP5_000894	Penicillin-binding protein 1A	*ponA*	0.0
SP5_001372	Penicillin-binding protein 2A	*pbp2A*	0.0
***Acid tolerance***			
SP5_002673	Sodium proton antiporter	*yvgP*	0.0
SP5_002530	ATP synthase subunit alpha	*atpA*	0.0
SP5_002526	ATP synthase subunit a	*atpB*	1.23e-162
SP5_002533	ATP synthase epsilon chain	*atpC*	1.88e-91
SP5_002532	ATP synthase subunit beta	*atpD*	0.0
SP5_002527	ATP synthase subunit c	*atpE*	2.57e-37
SP5_002528	ATP synthase subunit b	*atpF*	3.59e-80
SP5_002531	ATP synthase gamma chain	*atpG*	1.92e-211
SP5_002529	ATP synthase subunit delta	*atpH*	3.93e-116
SP5_000655	Decarboxylase	*yphJ*	1.07e-72
***Bile salt tolerance***			
SP5_002916	Linear amide C-N hydrolase, choloylglycine hydrolase family	−	2.38e-252
***Extreme temperature tolerance***			
SP5_001004	“Cold-shock” DNA-binding domain protein	*cspA*	3.08e-43
SP5_000711	Cold shock protein	*cspB*	4.62e-48
SP5_002482	Cold shock protein	*cspC*	6.22e-43
SP5_001854	Heat shock 40 kDa protein	*dnaJ*	9.45e-261
SP5_001853	Heat shock 70 kDa protein	*dnaK*	0.0
SP5_001695	Member of the small heat shock protein (HSP20) family	*hsp*	1.8e-99
SP5_000918	Member of the small heat shock protein (HSP20) family	*hsp1*	1.05e-111
SP5_001851	Negative regulator of class I heat shock genes (grpE- dnaK-dnaJ and groELS operons)	*hrcA*	1.6e-246
SP5_002404	Recovery of the cell from heat-induced damage, in cooperation with DnaK, DnaJ and GrpE	*clpC*	0.0
SP5_001504	Molecular chaperone	*GroEL*	0.0
SP5_001503	Co-chaperonin	*GroES*	1.7e-59
SP5_000774	Molecular chaperone	*clpB*	0.0
***Osmotic shock tolerance***			
SP5_001852	Response to hyperosmotic and heat shock	*grpE*	3.18e-127
SP5_000464	Glycine betaine	*gbuA*	9.19e-285
SP5_000465	Glycine betaine	*gbuB*	2.37e-188
SP5_000466	Glycine betaine	*opuAC*	3.51e-216
SP5_001064	Periplasmic glycine betaine choline-binding (lipo)protein of an ABC-type transport system (osmoprotectant binding protein)	*opuCC*	3.55e-222
SP5_002193	Periplasmic glycine betaine choline-binding (lipo)protein of an ABC-type transport system (osmoprotectant binding protein)	*choS*	6.97e-237
SP5_000112	Periplasmic glycine betaine choline-binding (lipo)protein of an ABC-type transport system (osmoprotectant binding protein)	*choS*	1.06e-112
***Oxidative stress survival***			
SP5_000337	Redox-regulated molecular chaperone	*hslO*	2.08e-208
SP5_002002	NADH dehydrogenase	*ndh*	0.0
SP5_000967	NADH oxidase	*nox*	0.0
SP5_001535	NADH oxidase	*nox*	0.0
SP5_001872	NADH oxidase	*npr*	0.0
SP5_002315	Member of the glutathione peroxidase family	*gpo*	9.78e-112
SP5_001166	Thiol-specific peroxidase	*tpx*	8.23e-117
SP5_001901	Peroxidase	*ywbN*	9.75e-228
***Cell wall formation***			
SP5_000209	Cell wall formation	*murA*	9.48e-300
SP5_001823	Polysaccharide biosynthesis protein	*ytgP*	6.15e-171
SP5_002246	Capsular exopolysaccharide family	*ywqD*	2.41e-143
SP5_002250	Glycosyltransferase like family 2	*epsIIG*	3.79e-89
SP5_002585	Glycosyltransferase like family 2	*epsG*	4.18e-151
SP5_002316	Glycosyl transferases group 1	*tagE3*	0.0
SP5_002317	Glycosyl transferases group 1	*tagE2*	0.0
SP5_002642	Glycosyl transferase family 8	*arbx*	1.17e-211
SP5_001889	Glycosyl transferase family 2	*ykcC*	8.07e-233
SP5_000560	D-alanine–D-alanyl carrier protein ligase	*dltA*	0.0
SP5_000558	D-alanyl carrier protein	*dltC*	6.97e-49
SP5_000557	Involved in the D-alanylation of LTA	*dltD*	8.29e-312
SP5_000561	D-Ala-teichoic acid biosynthesis protein	*dltX*	1.04e-27
***Adhesion capacity***			
***Putative adhesins***			
SP5_000853	Fibronectin-binding protein A (Fibronectin binding domain A)	*FbpA*	0.0
SP5_002267	Putative adhesin	*yvlB*	0.0
SP5_001633	Internalin J (MucBP domain)	*inlJ*	5.78e-287
SP5_002881	LPxTG domain protein (PillinD1 domain, SpaA domain, Ig-like fold)	−	1.25e-236
SP5_002694	Hydrolase, Collagen-binding protein	*mapA*	0.0
SP5_000963	NlpC P60 family protein (SlpA domain)	*p75*	7.12e-202
SP5_002397	CHAP domain protein (SibA CHAP domains)	*p40*	4.92e-201
***Moonlighting proteins***			
SP5_002227	Glycosyl hydrolase (LysM domain)	−	8.06e-232
SP5_000794	Leucine-rich repeat (LRR) protein (LysM domain)	−	1.68e-104
SP5_001575	Hydrolase (LysM domain)	−	1.64e-184
SP5_000723	Phosphoglycerate mutase	*pgm6*	4.69e-159
SP5_002706	Triosephosphate isomerase	*tpiA*	2.81e-180
SP5_000756	Elongation factor Tu	*tuf*	2.74e-285
***Supporting functions***			
SP5_000470	Sortase family protein	*srtA*	4.31e-166
SP5_002372	Sortase family protein	*srtA*	2.1e-143
SP5_002167	Sortase family protein	*srtB*	5.2e-188
SP5_002880	Sortase family protein	−	3.16e-258
***Biofilm formation***			
SP5_002245	Capsular polysaccharide biosynthesis protein	*epsB*	2.52e-169
SP5_000584	S-ribosylhomocysteine lyase	*luxS*	1.69e-112
SP5_000661	Transcriptional regulatory protein DesR	*desR*	8.42e-135
SP5_001469	Catabolite control protein A	*ccpA*	3.03e-232
SP5_000097	Cell envelope-like function transcriptional attenuator common domain protein	*brpA*	4.84e-256
SP5_000748	Competence protein ComEA	*comEA*	4.95e-146
SP5_000895	ComE operon protein 2	*comEB*	5.94e-111
SP5_002282	Competence protein	*comFC*	1.57e-106
SP5_002283	Helicase C-terminal domain protein	*comFA*	1.58e-301
SP5_002653	Type II secretion system	*comGB*	1.96e-194

Genes conferring resistance to the acidic pH of the stomach, bile salts and digestion enzymes were, also, annotated in the genome of *Lc. paracasei* SP5, using interconnected approaches. Specifically, 11 loci coding for acid tolerance proteins were identified (Table 3). These include a complete cluster for F0-F1 ATPase proton pump, that regulates cytoplasmic pH by pumping out H^+^ after ATP hydrolysis (Deckers-Hebestreit and Altendorf, 2003), as well as a sodium: proton antiporter for sodium and pH homeostasis (Huang et al., 2016). I t should be noted that acid tolerance can be manifested in a plethora of ways, including cell wall modifications and biofilm formation (Liu et al., 2021). In this context, biofilm formation supports survival and proliferation in hostile environments, as viable bacteria are protected in a polysaccharidic capsule (Liu et al., 2021). Genes implicated in biofilm formation, such as *luxS* and *comC*, *comD* and *comE*, were also annotated. Molecular chaperones and co-chaperons, as well as repair mechanisms, including ultraviolet (UV) excinuclease gene (*uvrA*) and RecA-assisted DNA repair, also found in the genome of *Lc. paracasei* SP5, could support survival in acidic conditions (Table 3; Thompson and Blaser, 1995; Hanna et al., 2001). Concerning resistance to bile acids, *Lc. paracasei* SP5 codes for a linear amide C-N hydrolase belonging to the choloylglycine hydrolase family. Proteins of this family were previously shown to neutralize bile acids *via* deconjugation (Begley et al., 2006). No other bile salt hydrolases were identified, however, phenotypic predictions and previous experimental data show that the strain is resistant to bile (Supplementary Figure 5; Mantzourani et al., 2019a).

The probiotic character also includes sensitivity to common antibiotics and lack of virulence genes. In this context, the strain does not possess acquired genes conferring antimicrobial resistance. However, it harbors a genetic cluster involved in vancomycin resistance. Vancomycin specifically binds to the D-alanine/D-alanine terminus of the muramyl pentapeptide of peptidoglycan precursors, inhibiting its polymerization and, thus, cell wall formation. Genes conferring resistance to this antibiotic usually regulate the production of different peptidoglycan precursors. The genome of *Lc. paracasei* SP5 carries the resistance genes *vanR* and *vanZ*. The mechanism of action of VanZ remains unknown, however, there are indications that it can exclude vancomycin and other lipoglycopeptide antibiotics from the cell wall, increasing the minimum inhibitory concentration required (Vimberg et al., 2020). VanR is a response element that activates transcription of the *vanHAX* cluster after vancomycin exposure. These genes are chromosomally encoded, and thus, they cannot participate in events of horizontal gene transfer. Vancomycin resistance is a shared characteristic of lactobacilli that does not raise safety concerns (Gueimonde et al., 2013). It is important to note, however, that vancomycin resistance is not intrinsic in all LAB strains. The most characteristic example is that of *Enterococcus* strains that, although present a wide spectrum antimicrobial activity and potential probiotic attributes, do not possess the Generally Recognized as Safe (GRAS) or Qualified Presumption of Safety (GPS) status (Ricci et al., 2017) due to the fact that they harbor transferable vancomycin resistance genes and virulence factors (Hanchi et al., 2018). Whole genome analysis of *Lc. paracasei* SP5 with PathogenFinder and VirulenceFinder algorithms did not reveal any homologous genes to virulence factors of common, clinically relevant pathogens. Additionally, the Traitar tool predicted that the strain does not possess beta hemolytic or coagulase activity (Supplementary Figure 5). Antimicrobial capacity is one of the most well-studied attributes of potentially probiotic strains (Silva et al., 2020). To this aim, BAGEL4 analysis revealed the presence of five regions of interest for bacteriocin production in the genome *Lc. paracasei* SP5 (Supplementary Figure 10). Specifically, these genetic clusters encode the production of Enterolysin A (Class III bacteriocin), carnocine CP (putative class II bacteriocin) and Enterocin X beta chain (Class IIc bacteriocin, circular peptide), suggesting the potential antimicrobial capacity of the strain. Furthermore, the lantibiotic transporter LanT (Singh and Sareen, 2014) was identified in the genome of the strain, indicating the possible presence of novel clusters for lantibiotic production. In this context, other *Lc. paracasei* strains capable of producing functional bacteriocins in a plethora of matrixes, where found active against gram negative strains, such as *Escherichia coli* (Belguesmia et al., 2020; Madi-Moussa et al., 2022), gram positive bacteria, including *Staphylococcus aureus* (Jiang et al., 2022), as well as food spoilage microbiota (Zhu et al., 2021). Interestingly, previous findings indicate that, *Lc. paracasei* SP5 can present antibacterial and antifungal activity *in situ* (Plessas et al., 2021). In that sense, comprehensive characterization of the antimicrobial potential of the strain is currently being undertaken.

### Genome-Wide Analysis and *in vitro* Study of the Adhesive Phenotype of *Lc. paracasei* SP5

**FIGURE 6:**
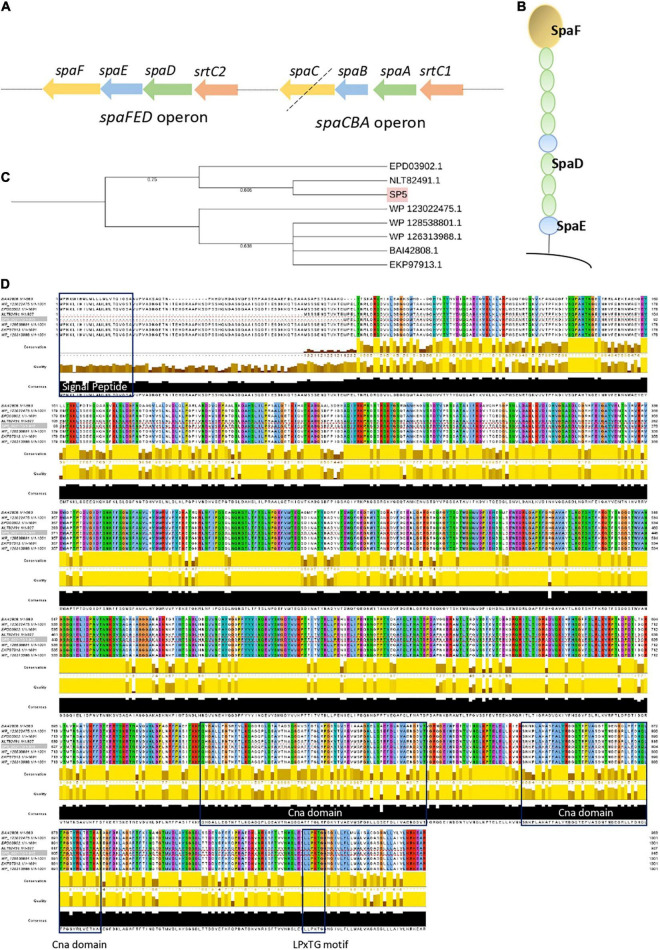
Analysis of the *spaCBA* and *spaFED* cluster encoded in the genome of *Lc. paracasei* SP5. **(A)** Graphical depiction of the *spaCBA* and *spaFED* and pili clusters annotated by PGAP in the genome of *Lc. paracasei* SP5. The black dotted line signifies that *spaC* is a pseudogene. **(B)** Graphical depiction of the *spaFED* pilus. **(C)** Neighbor-Joining phylogenetic tree of *Lc. paracasei* (EPD03902.1, NLT82491.1, WP_123022475.1, WP_128538801.1, WP_126313988.1) and *Lc. rhamnosus* GG (BAI42808.1) putative *spaF* gene sequences. Gene alignment was performed using ClustalW and the tree was constructed on the iTol server. Highlighted in pink is the *spaF* gene sequence of *Lc. paracasei* SP5 **(D)** Visualization of *spaF* alignment was performed using Jalview. Blue boxes indicate the N-terminal signal peptide, two Cna domains (669–735 aa, 764–818 aa), and a C-terminal LPxTG motif.

Adhesins carrying motifs for exposure on the bacterial surface (LPxTG) and for specific interactions with host receptors, glycosylated proteins and polysaccharides of the gut niche and extracellular matrix (WxL) were annotated using PGAP and studied further using InterPro, Pfam and ScanProsite. Amongst the predicted proteins, genes coding for fibronectin-binding protein A (*fbpA*), two mucin binding proteins; Internalin J (*inlJ*), and the mucus adhesion promoting protein (*mapA*) were annotated in the genome sequence (Table 3). Interestingly, proteomic studies have revealed that bile acids and heat stress resulted in their overexpression in *Lc. paracasei* species, leading to increased adhesion ability (Bengoa et al., 2018; Adu et al., 2020). Several sortase genes (*srtA*, *strB*, *srtC1*, *srtC2*) were found in the genome of *Lc. paracasei* SP5. Sortases play an important role in the maturation and exposure of LPxTG motif-carrying proteins on the cell wall, supporting the adhesive ability of strains. Furthermore, the presence of *spaCBA* or *spaFED* pili was estimated using BLASTp locally, using as template sequences derived from LGG, one of the first lactobacilli shown to produce these pili (Reunanen et al., 2012). *Lc. paracasei* SP5 codes for *spaA*, *spaB* and *srtC1*, however, the adhesin *spaC* is missing (pseudogene), suggesting that the pilus may not be functional (Figure 6A). Furthermore, its genome contains a full cluster for the production of SpaFED pili (Figures 6A,B), that presents more than 77 % similarity to that of LGG, according to BLASTp analysis. Further bioinformatic analysis on the sequence of the SpaFED pilus adhesin, *spaF* revealed that it presents high similarity (> 99 %) to cell wall proteins encoded by other lactobacilli, showing a close phylogenetic relationship with an LPXTG cell wall anchor domain-containing protein, derived from *Lc. paracasei* subsp. *paracasei* isolate AS01afH2WH_17 (Figure 6C). It has a length of 915 aa, and a molecular weight of approximately 10 kDa. Furthermore, SpaF has an acidic isoelectric point (pI) of 5.18, and ProtParam categorizes it as stable. Domain and motif analysis revealed that it contains two Cna-B domains that enclose a collagen adhesive domain, and a C-terminal LPxTG (LPKTG) motif (Figure 6D). It is important to note that the sequence is lacking a N-terminal signal peptide and thus, questions about its successful incorporation on the bacterial surface are being raised. However, THMM 2.0 analysis showed that the first 887 aa may be exposed on the cellular surface, while a transmembrane region including the LPxTG domain, and an intercellular domain were identified in the remaining amino acids. The *spaCBA* cluster plays a significant role in the adhesive capacity of the *Lacticaseibacillus* genus and was previously identified in LGG (Reunanen et al., 2012), *Lc. casei* LOCK 0919 (Aleksandrzak-Piekarczyk et al., 2016) and *Lc. paracasei* LP10266 (Tang et al., 2021), among others. The *spaFED* cluster is not as widespread as the *spaCBA* genetic locus; however, it is detected in most *Lc. rhamnosus* strains, as well as in several *Lc. paracasei* strains (Broadbent et al., 2012). It is important to note, that the regulatory factors triggering its expression remain elusive (Rintahaka et al., 2014), while the crystal structures of the pilins have only recently been solved (Megta et al., 2019). Therefore, visualization of these structures on *Lc. paracasei* SP5 and the comprehensive characterization of their physicochemical properties are required to better understand their contribution to the adhesion capacity of the strain.

Moonlighting proteins with putative adhesive properties were also identified in the genome (Table 3). These include the chaperone GroEL and the co-chaperonin GroES, as well as glycolytic enzymes, such as triosephosphate isomerase (*tpiA*) and glyceraldehyde 3-phosphate dehydrogenase (GAPDH), that under appropriate conditions can be exposed in the cellular surface and participate in the adhesion phenotype of strains (Jeffery, 2019). Studies have shown that availability of carbon sources, plant polyphenols and prebiotic fiber could influence their exposure on the cell surface, and subsequently the adhesion capacity of strains (Jeffery, 2019). Concerning the host factors that moonlighting proteins bind to, GAPDH was shown to interact with host mucin (Patel et al., 2016), TpiA with laminin (Pereira et al., 2007) and GroEL with plasminogen (Hagemann et al., 2017) and mucin (Bergonzelli et al., 2006). Interestingly, pathogens utilize moonlighting proteins to colonize the host’s epithelia, and thus probiotics can compete with them for binding sites (Jeffery, 2018). More specifically, probiotics that code for homologous surface proteins with pathogenic species can effectively block their adhesion in the gut niche (van Zyl et al., 2020). In this context, the exclusion of *Listeria monocytogenes* EGDe by *Lp. plantarum* 423 *in vivo*, was attributed to the presence of the *mapA* gene (van Zyl et al., 2019), also annotated in the genome of *Lc. paracasei* SP5. Similar observations were made, for other lactobacilli proteins, such as S-layer proteins and sortase-dependent cell surface proteins (SDPs; van Zyl et al., 2020). Furthermore, non-protein macromolecules, such as exopolysaccharides can also mediate the adhesive phenotype (Alp and Kuleaşan, 2019). In this light, genes for exopolysaccharide biosynthesis and transport were pinpointed in the genome of *Lc. paracasei* SP5 (Table 3). Concerning, microbe-microbe adhesive interactions *Lc. paracasei* SP5 harbors genes participating in autoaggregation, biofilm production and LuxS signaling (Table 3). Proteins with moonlighting functions, pilins and the capsular polysaccharides of the cellular surface may also contribute to these phenotypes, as previously described (Nwoko and Okeke, 2021).

**FIGURE 7:**
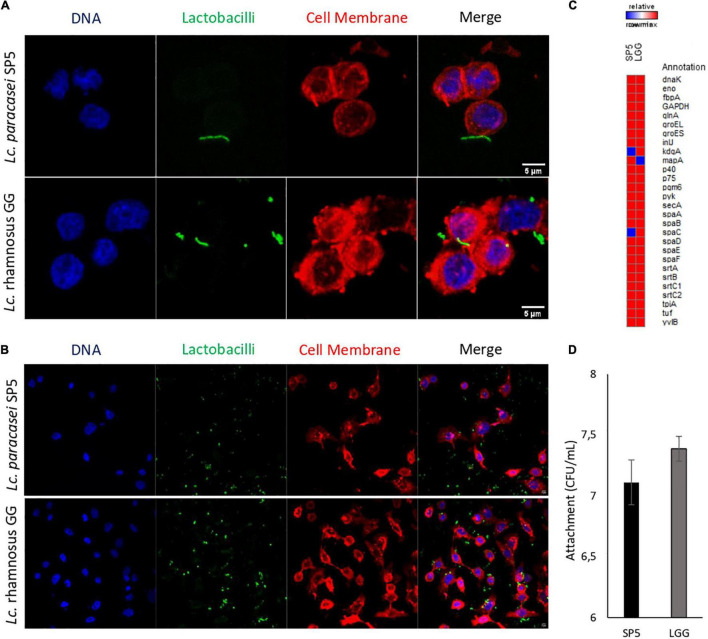
Evaluation of the adhesion capacity of *Lc. paracasei* SP5 onto the human colon cancer adenocarcinoma cell line, HT-29. **(A,B)** Representative photos from confocal fluorescent microscopy showing the adhesion capacity of *Lc. paracasei* SP5 or *Lc. rhamnosus* GG. Panel A represents a zoomed in image of a 40x photo, edited by ImageJ (version 1.53f51), while panel B represents a photo of the original magnification (40×). Bacteria are stained with CFSE (green), eukaryotic nuclei (blue) and cell membranes (red) are stained with Hoescht and CellBrite Red Cytoplasmic Membrane Dye, respectively (scale bar, 5 μm). **(C)** Gene matrix depicting the presence or absence of adhesion-related proteins in the genomes of *Lc. paracasei* SP5 and *Lc. rhamnosus* GG, annotated by PGAP. **(D)** Determination of attached bacterial counts (CFU/mL) of *Lc. paracasei* SP5 (black bar) or *Lc. rhamnosus* GG (grey bar) after 4 h co-incubation with HT-29 monolayers. Results are presented as mean ± standard deviation.

The adhesive properties of the strain were further investigated *in vitro* using a microbiological method and confocal microscopy. More specifically, it was shown that *Lc. paracasei* SP5 can bind to HT-29 monolayers with comparable efficiency to LGG (Figure 7). LGG was used as a reference strain, due its well-characterized adhesive properties (Rasinkangas et al., 2020). Confocal microscopy provided visual evidence of these interactions and was used to determine the percentage of cells carrying adhered bacteria (Figures 7A,B). More specifically, *Lc. paracasei* SP5 adhered to 231 out of total 1575 cells (14.7%), while LGG adhered to 464 out of total 1195 cells (38.3%). Further comparative bioinformatic analysis revealed that both strains present a very similar profile in terms of the production of adhesins and adhesion-related proteins (Figure 7C). Many LAB strains present adhesion capacity *in vitro*, however, when in the gut niche, probiotics can attach to the mucus and epithelium with varying degrees of success, only leading to transient gut colonization (Monteagudo-Mera et al., 2019). Thus, studies on more sophisticated models of the gut or in animals could provide better insights into the behaviour of *Lc. paracasei* SP5 in physiological conditions.

## Conclusion

In this work, whole genome sequencing, annotation and comprehensive bioinformatic analyses were utilized to further characterize the probiotic and biotechnological potential of *Lc. paracasei* SP5, a strain originally isolated from kefir grains. Comprehensive phylogenomic analysis confirmed the classification of the novel strain to the *Lc. paracasei* species. Concerning genome stability and safety, *Lc. paracasei* SP5 does not harbor mobile elements, acquired antimicrobial resistance genes or virulence factors. Genome annotation and functional characterization revealed that the strain can utilize a plethora of carbohydrates as energy sources, supporting its nomadic character. Furthermore, it was found that *Lc. paracasei* SP5 codes for several factors mediating survival during food processing and GI passage, as well as for microbe-host interactions. Indeed, it possesses genes implicated in the production of proteins and polysaccharides that participate in mucosa and epithelium attachment, and subsequently the adhesion capacity of the novel strain was validated *in vitro*. It was shown that, *Lc. paracasei* SP5 adheres to cell monolayers with comparable efficiency to LGG. These findings suggest that, *Lc. paracasei* SP5 is a good probiotic candidate, with capacity to be incorporated in novel fermented food products, providing fertile ground for biotechnological innovation. Further studies, including metabolomic and proteomic approaches, to characterize the strain at multiple levels, will provide a more complete view of its mechanisms of action and health-promoting properties.

## Data Availability Statement

The data presented in this study are deposited in the DDBJ/ENA/GenBank repository, accession number JAKJPP000000000. The version described in this manuscript is JAKJPP010000000.

## Author Contributions

PK, MK, and AG designed the study. DK, CE, KT, and IM carried out the experiments. DK, KT, AA, PK, and MK analysed the data. DK, CE, and AG participated in the writing of the manuscript. AA, SP, PK, MK, and AG contributed to editing and critical reviewing of the manuscript. AA, SP, MK, and AG took charge of the resources. All authors had read and approved the final manuscript.

## Conflict of Interest

The authors declare that the research was conducted in the absence of any commercial or financial relationships that could be construed as a potential conflict of interest.

## Publisher’s Note

All claims expressed in this article are solely those of the authors and do not necessarily represent those of their affiliated organizations, or those of the publisher, the editors and the reviewers. Any product that may be evaluated in this article, or claim that may be made by its manufacturer, is not guaranteed or endorsed by the publisher.
