# Supplementary figures and images for: Genomic Insight Into Lacticaseibacillus paracasei SP5, Reveals Genes and Gene Clusters of Probiotic Interest and Biotechnological Potential
Source: Front Microbiol. 2022 Jun 16;13:922689. doi: 10.3389/fmicb.2022.922689 (PMC9244547; doi:10.3389/fmicb.2022.922689)

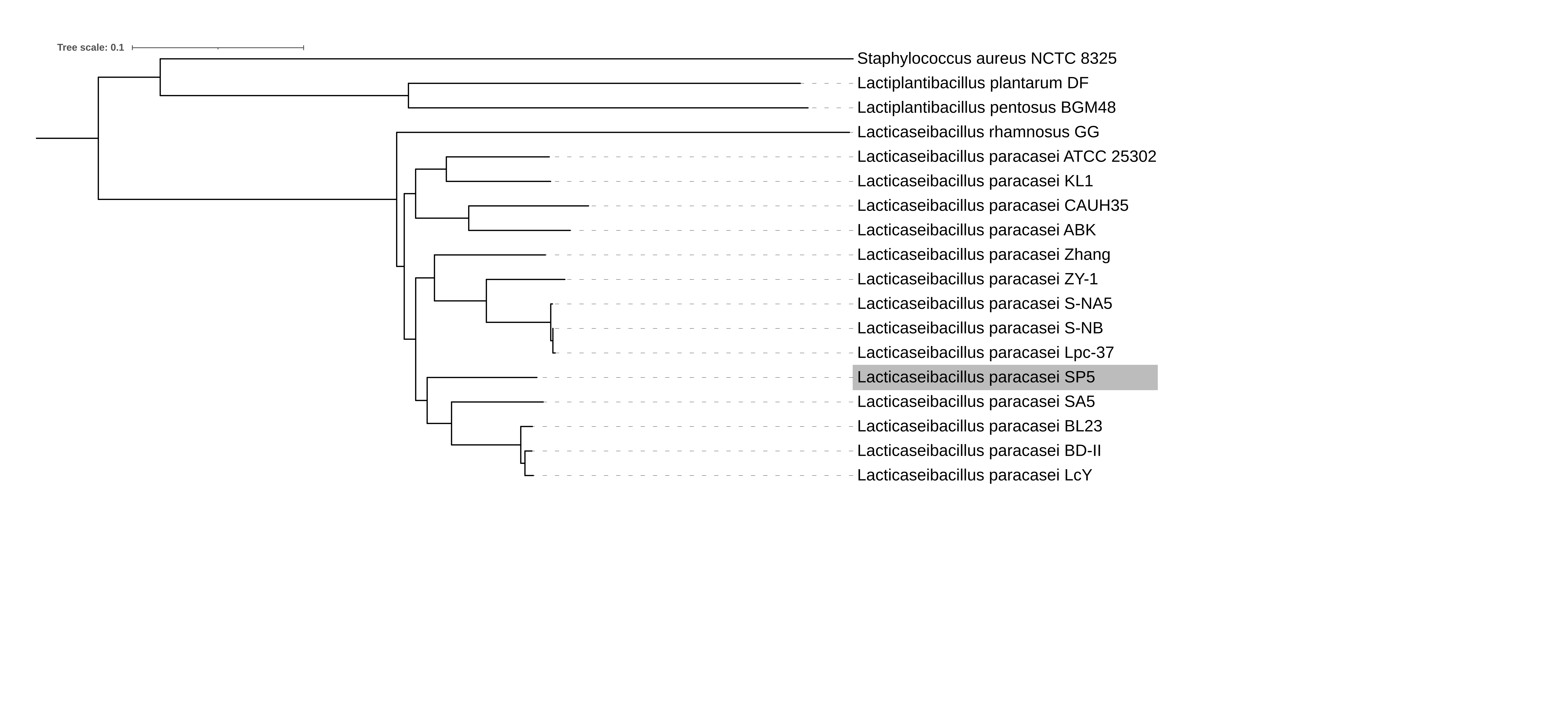

Supplement: Supplementary file 1 [file Image_1.PNG]

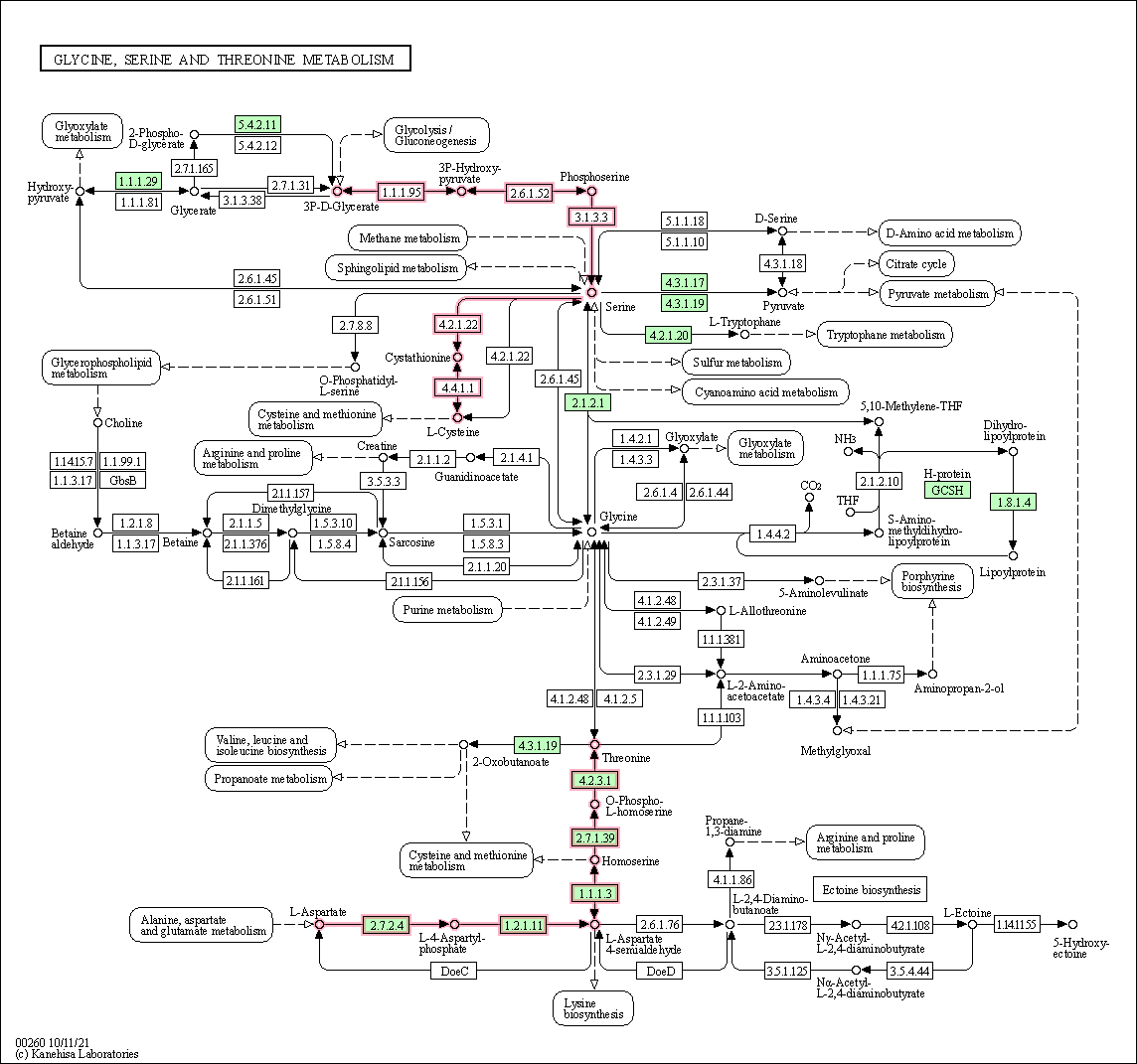

Supplement: Supplementary file 2 [file Image_2.PNG]

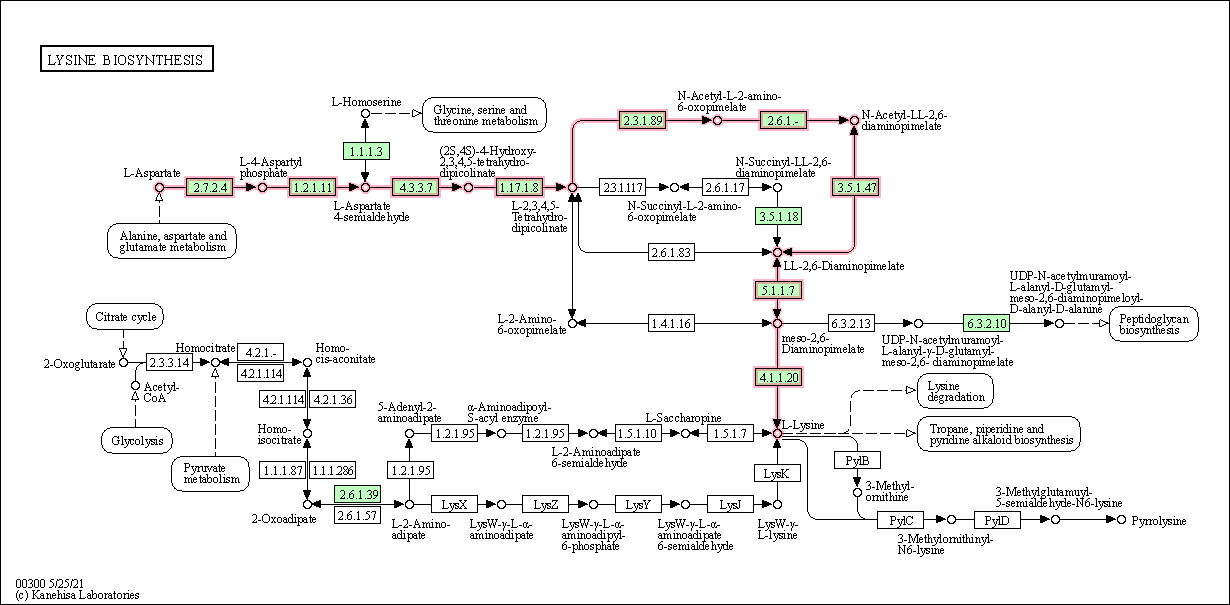

Supplement: Supplementary file 3 [file Image_3.PNG]

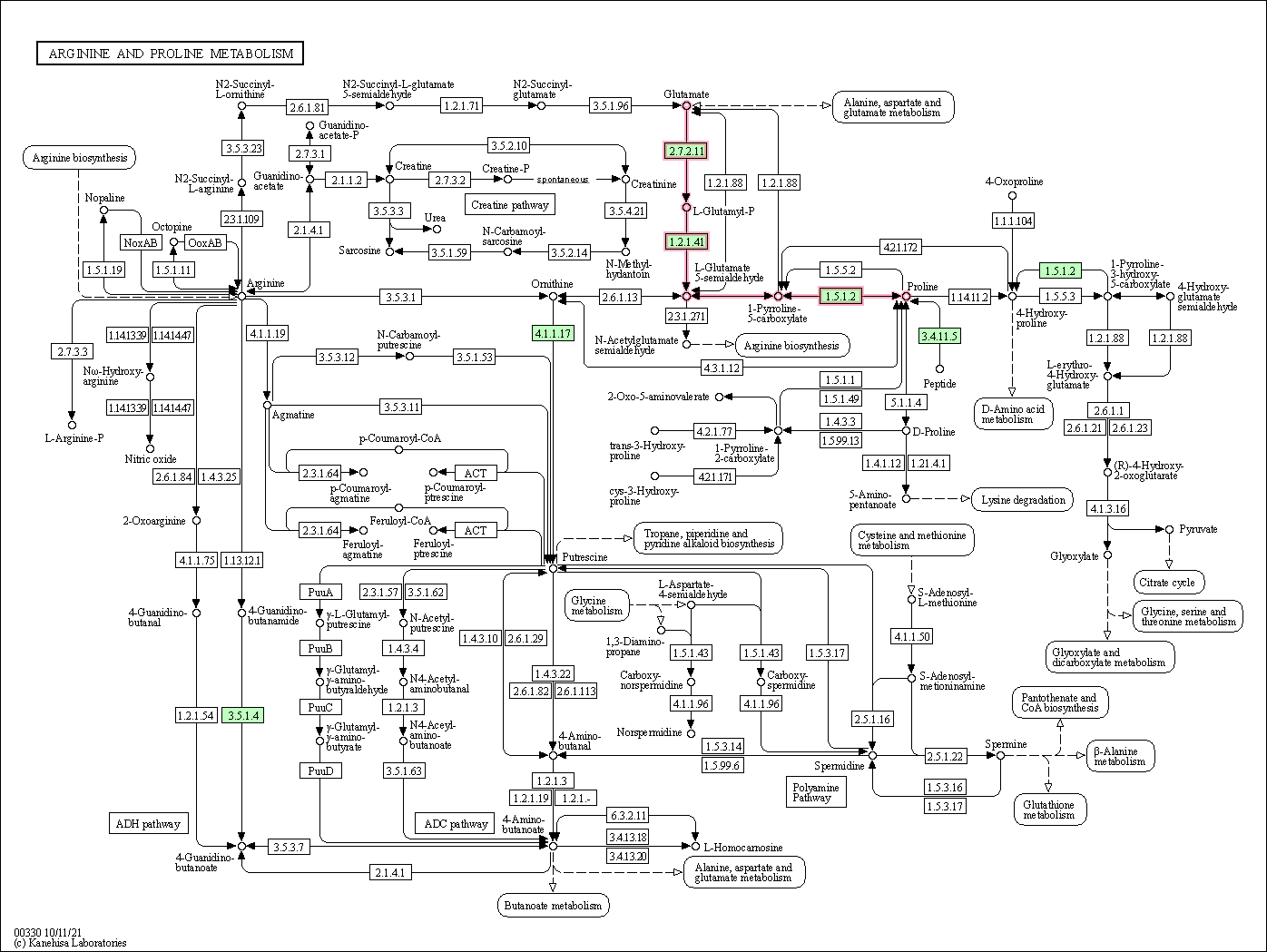

Supplement: Supplementary file 4 [file Image_4.PNG]

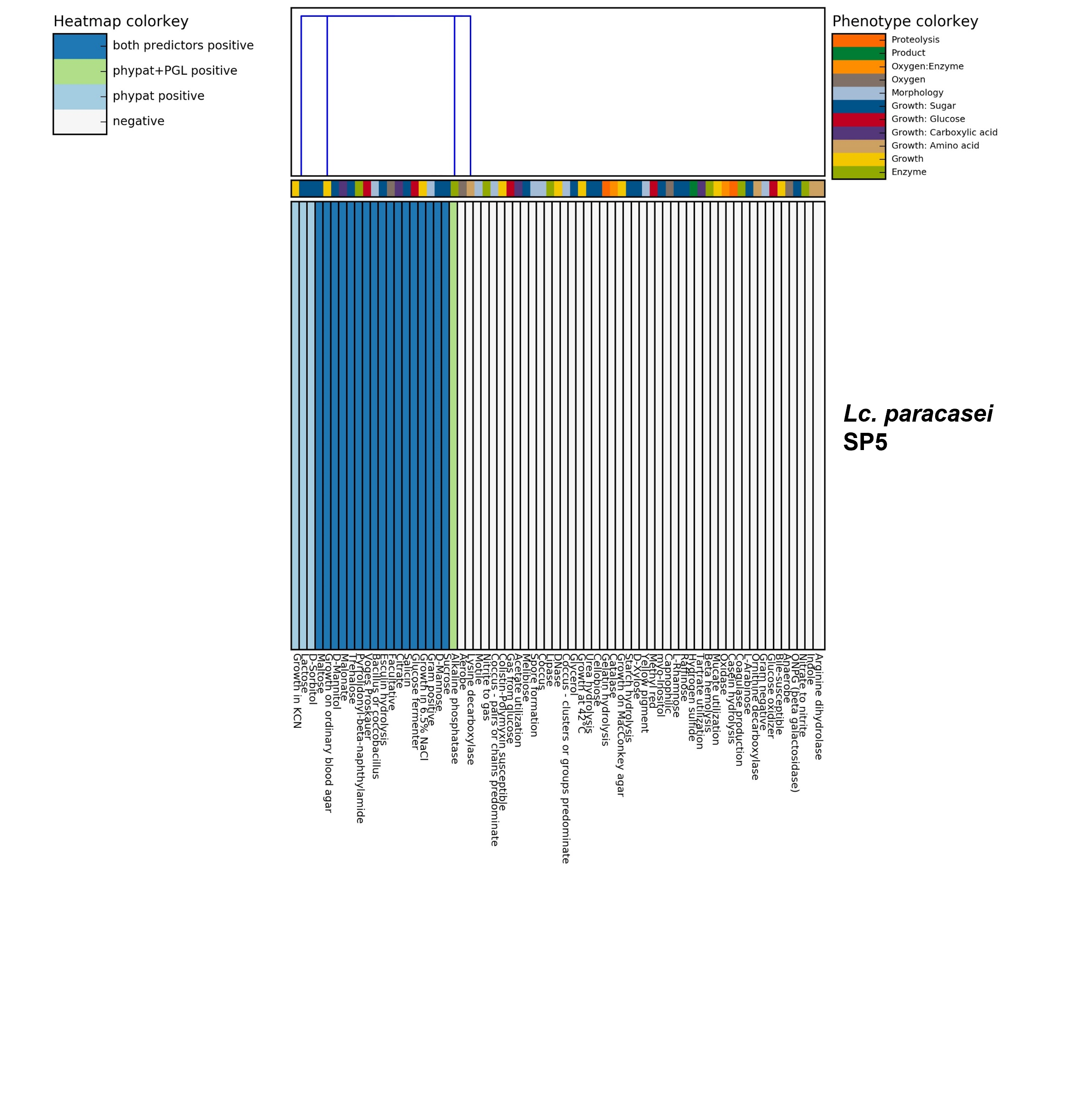

Supplement: Supplementary file 5 [file Image_5.PNG]

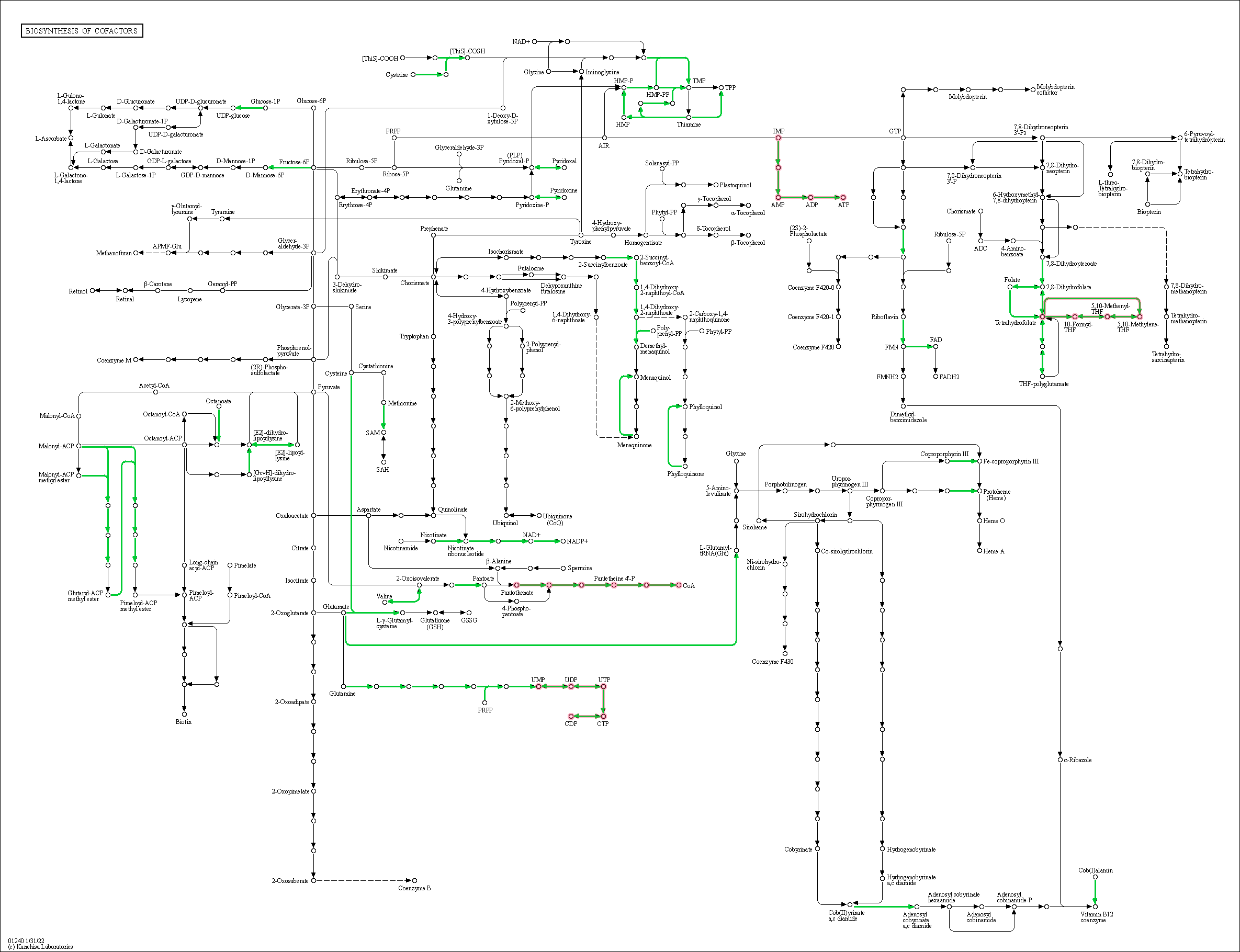

Supplement: Supplementary file 6 [file Image_6.PNG]

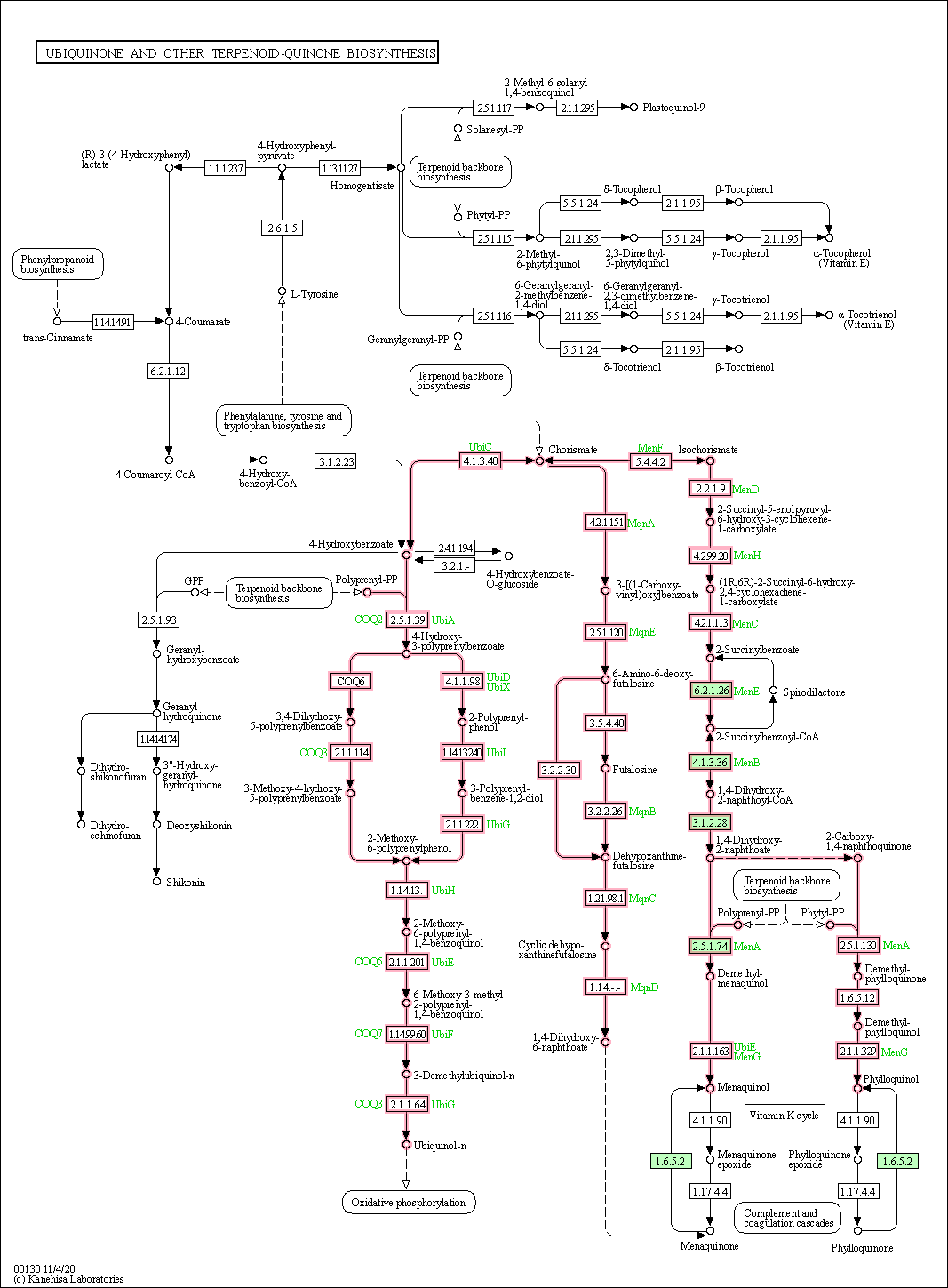

Supplement: Supplementary file 7 [file Image_7.PNG]

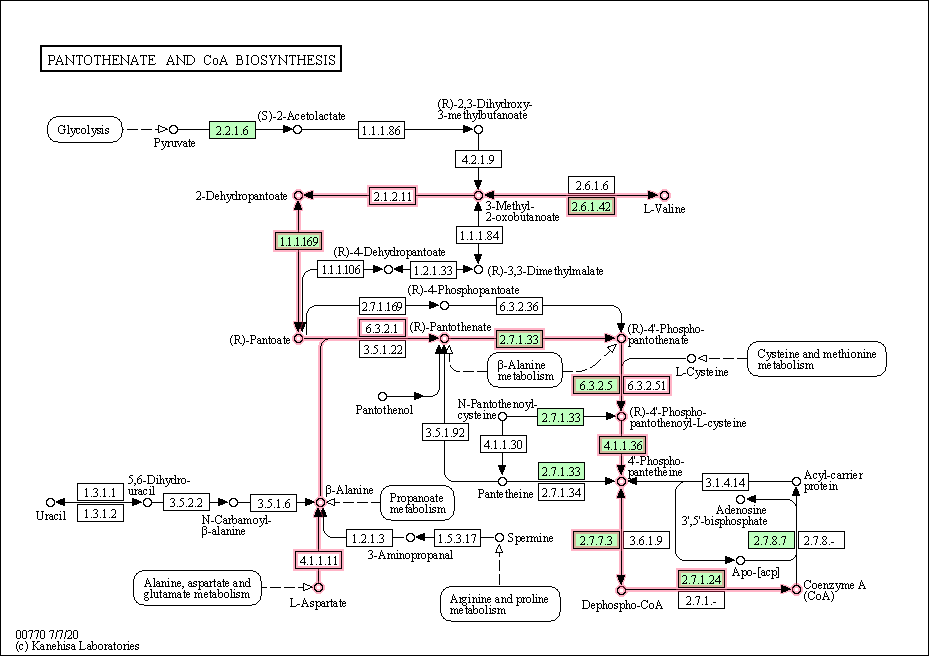

Supplement: Supplementary file 8 [file Image_8.PNG]

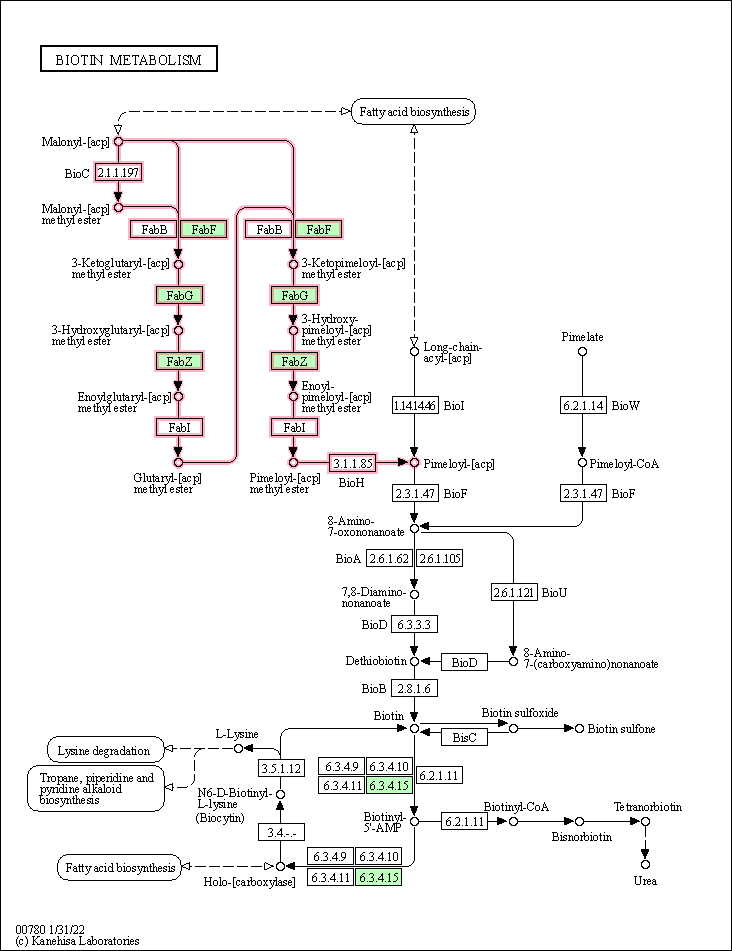

Supplement: Supplementary file 9 [file Image_9.PNG]

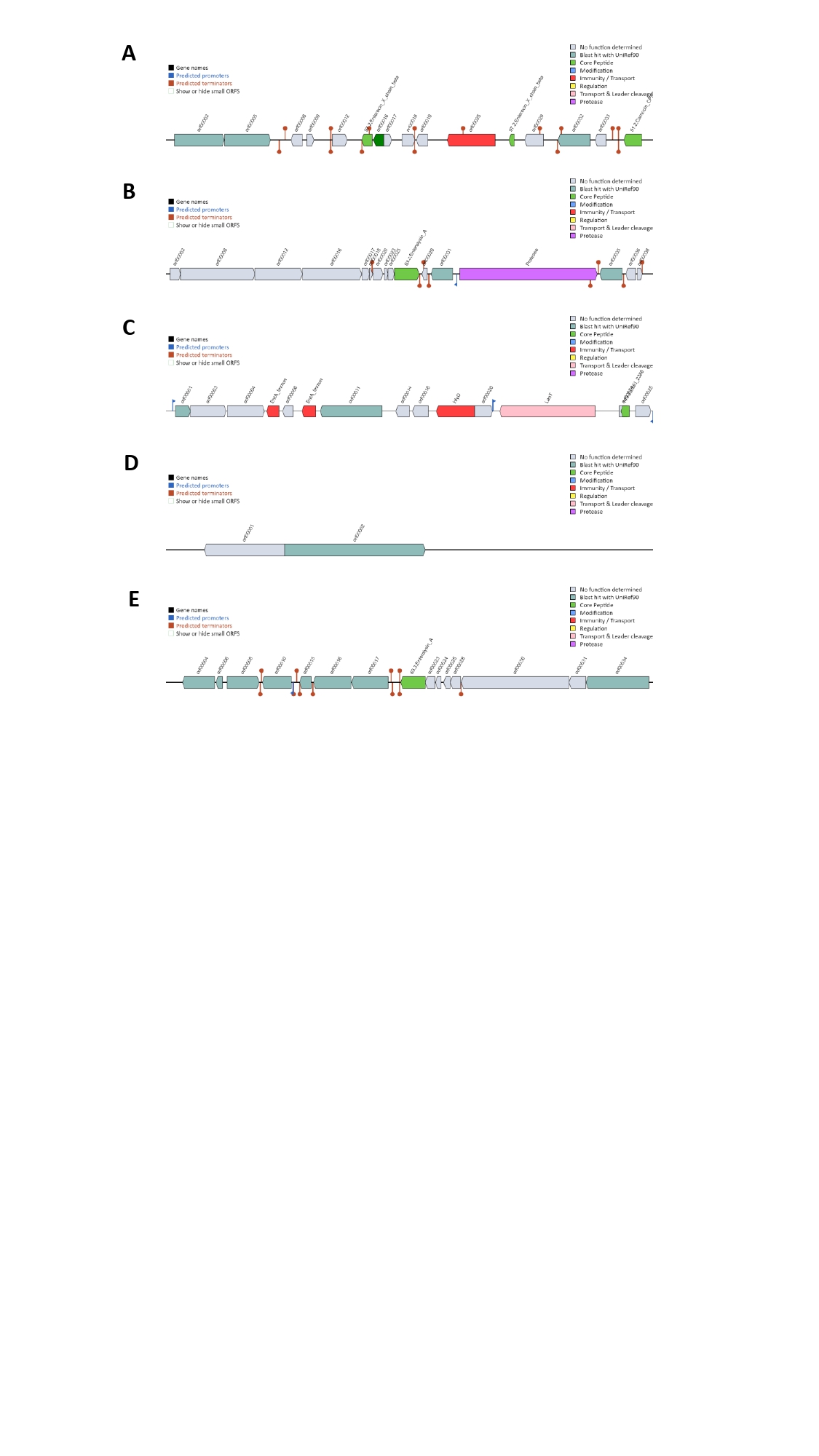

Supplement: Supplementary file 10 [file Image_10.JPEG]
