# Supplementary material for: Genomic Insight Into Lacticaseibacillus paracasei SP5, Reveals Genes and Gene Clusters of Probiotic Interest and Biotechnological Potential
Source: Front Microbiol. 2022 Jun 16;13:922689. doi: 10.3389/fmicb.2022.922689 (PMC9244547; doi:10.3389/fmicb.2022.922689)
Supplement: Supplementary file 16 [file Data_Sheet_1.PDF]

Supplementary Figure 1. Phylogenetic tree based on whole genome sequences of of 14 *Lc. paracasei* strains (including *Lc. paracasei* SP5), *Lc. rhamnosus* GG, *Lactiplantibacillus pentosus* BGM48 and *Lactiplantibacillus plantarum* DF. *Staphylococcus aureus* NCTC 8325 was used as an outgroup. Whole genome alignment was performed using ProgressiveMauve and the phylogenetic tree was constructed on iTol.

Supplementary Figure 2. *Lc. paracasei* SP5 contains a full pathway for the production of Threonine, as evidenced by KEGG pathway analysis. Serine and threonine biosynthetic clusters are denoted in red, while proteins encoded by *Lc. paracasei* SP5 are marked green.

Supplementary Figure 3. *Lc. paracasei* SP5 contains a full pathway for the production of Lysine, as evidenced by KEGG pathway analysis. The biosynthetic cluster is denoted in red, while proteins encoded by *Lc. paracasei* SP5 are marked green.

Supplementary Figure 4. *Lc. paracasei* SP5 contains a full pathway for the production of Proline, as evidenced by KEGG pathway analysis. The biosynthetic cluster is denoted in red, while proteins encoded by *Lc. paracasei* SP5 are marked green.

Supplementary Figure 5. Phenotypic analysis of the novel *Lc. paracasei* strain SP5, based on predictions made by Traitar.

Supplementary Figure 6. *Lc. paracasei* SP5 contains pathways for the production of co-factors as evidenced by KEGG pathway analysis (denoted as green).

Supplementary Figure 7. *Lc. paracasei* SP5 codes for ubiquinone and terpenoid-quinone biosynthetic clusters. The biosynthetic clusters are denoted in red, while proteins encoded by *Lc. paracasei* SP5 are marked green.

Supplementary Figure 8. *Lc. paracasei* SP5 codes for pathothenate and CoA biosynthetic clusters. The biosynthetic clusters are denoted in red, while proteins encoded by *Lc. paracasei* SP5 are marked green.

Supplementary Figure 9. Pathways coded in the genome of *Lc. paracasei* SP5 for biotin metabolism. The pathways are denoted in red, while proteins encoded by *Lc. paracasei* SP5 are marked green.

Supplementary Figure 10. Annotation of areas of interest (AOI) containing putative bacteriocin clusters performed by BAGEL4. Five AOI (A-E) were found in the genome of *Lc. paracasei* SP5.
